# Correction: The rhizosphere of *Phaseolus vulgaris* L. cultivars hosts a similar bacterial community in local agricultural soils

**DOI:** 10.1371/journal.pone.0325522

**Published:** 2025-06-02

**Authors:** Griselda López Romo, Rosa Isela Santamaría, Patricia Bustos, Francisco Echavarría, Luis Roberto Reveles Torres, Jannick Van Cauwenberghe, Víctor González

There are errors in the Funding statement. The correct Funding statement is as follows: PAPIIT-UNAM IN208021 for VG supported this study. GLR is a doctoral student from Programa de Doctorado en Ciencias Biomédicas, Universidad Nacional Autónoma de México (UNAM), and received a fellowship (No. CVU 746216) obtained from CONAHCYT. J.V.C. was also supported by the Deutsche Forschungsgemeinschaft (DFG, German Research Foundation) under Germany’s Excellence Strategy–EXC 2051–Project-ID 390713860 and by the European Research Council (ERC) Consolidator grant 865694: DiversiPHI, the Deutsche Forschungsgemeinschaft (DFG, German Research Foundation) under Germany’s Excellence Strategy–EXC 2051–Project-ID 390713860, and the Alexander von Humboldt Foundation in the context of an Alexander von Humboldt-Professorship founded by German Federal Ministry of Education and Research. This research was supported by the PAPIIT-UNAM IN208021 for VG. The funders had no role in study design, data collection and analysis, decision to publish, or preparation of the manuscript.
