# Supplementary material for: The rhizosphere of Phaseolus vulgaris L. cultivars hosts a similar bacterial community in local agricultural soils
Source: PLoS One. 2025 Mar 20;20(3):e0319172. doi: 10.1371/journal.pone.0319172 (PMC11925306; doi:10.1371/journal.pone.0319172)
Supplement: S1 Supporting methods — (PDF) [file pone.0319172.s001.pdf]

Supporting methods.

The rhizosphere of *Phaseolus vulgaris* L. cultivars hosts a similar bacterial community in local agricultural soils.

Griselda López-Romo[1], Rosa Isela Santamaría[1 ¶], Patricia Bustos[1¶], Francisco Echavarría[2], Luis Roberto Reveles-Torres[2], Jannick Van Cauwenberghe[3], and Víctor González[1, *]

[1] Centro de Ciencias Genómicas, Universidad Nacional Autónoma de México, Cuernavaca, Morelos, Mexico.

[2] Instituto Nacional de Investigaciones Forestales, Agrícolas y Pecuarias, Calera, Zacatecas, Mexico.

[3] Institute of Biodiversity, Faculty of Biological Sciences, Cluster of Excellence Balance of the Microverse, Friedrich Schiller University Jena, Jena, Germany.

Database choice and Kraken2 confidence score evaluation.

In this study, we aimed to capture a broad range of genomic sequences to determine the diversity of the bacterial taxa. We utilized Kraken2 in our analysis with the PlusPFP database because of its robustness for broad-spectrum taxonomic classification of microbiomes (1-3). PlusPFP encompasses a wide range of microorganisms, expanding upon the Standard Database, which includes archaeal, bacterial, viral, and human sequences from NCBI RefSeq. Furthermore, PlusPGFP incorporated supplementary reference sequences for protozoa, fungi, and plants. We anticipated covering and discarding contaminant sequences from both plants and humans. Additionally, we expected sequence information for eukaryotes (fungi) and viruses; however, they were poorly represented in the metagenomes (see response 2 below) and were not utilized in our analysis. Conversely, the bacterial domain exhibited the highest number of sequences reads and diversity in metagenomes (S2 Table; S3 to S5 Figs.)

Several studies have addressed the performance of Kraken2 using different confidence parameters in simulated and mock databases of various sizes (2, 4). Kraken2 demonstrates efficacy in classifying sequence reads using a large database, similar in size to PlusPFP, at a

default confidence score of zero (4). At this confidence score, a satisfactory recall of taxa remained, approximating the optimum of 0.69 (with a confidence score of 0.10). This indicates that these parameters accurately identified true-positive taxa. However, at a default score of zero, the overall performance of F1 is considered suboptimal (4).

Considering these parameters, we evaluated the effect of the default confidence score (zero) and three more stringent confidence score parameters (0.2, 0.4, and 0.6) on a sample of metagenomes derived from the bulk soil and rhizosphere. A consistent decrease in the number of classified sequence reads was observed as the confidence threshold increased from the default value of 0 to 0.6, accompanied by a corresponding increase in unclassified reads (S4 Fig.). In both the bulk soil and rhizosphere, the classified reads assigned to species diminished substantially, along with ~~the~~ diversity parameters (S5 and S6 Figs.). The Shannon index also exhibited low values at a strict confidence level threshold (S6 Fig). Furthermore, the effect on classified reads was more pronounced in bulk soil than in the rhizosphere. This result led us to conclude that stringent confidence levels did not capture the full diversity of the sample, and significant taxa could be excluded because of their low representation, such as in bulk soil. Consequently, we opted to utilize the default Kraken2 parameters and subsequently examined the changes in abundance between the soil and rhizosphere to assess whether poorly represented taxa in the soil transitioned to abundance in the rhizosphere.

In the second instance, we evaluated the performance of Kraken2 using ribosomal RNA gene databases, specifically GreenGenes and RDP (5). In S7 Fig. shows comparisons of taxonomic assignments made using Plus-PFP, GreenGenes, and RPD databases. A total of 55% (808/1464) of the genera classified using Kraken2-PlusPFP were shared with the Kraken2-RDP database (S7 Fig.). Conversely, the number of classified genera in the GreenGenes database was less than that with RDP and shared approximately 38% (564/1464) with the classification with Kraken2-PlusPFP. Furthermore, the classifications with RDP were sparsely distributed among the metagenomes, in contrast to the homogeneity observed in the distribution of the Kraken 2-PlusPFP assignments across all 29 metagenomes (S7 Fig.).

Therefore, we concluded that the use of Kraken2 with the PlusPFP database provides a robust and diverse taxonomic classification for metagenomic sequences, especially in the bacterial domain. However, the application of stricter confidence parameters significantly reduces the taxonomic diversity detected, which may exclude important taxa with low representation, as observed in soil samples. Therefore, the default parameters of Kraken2 were preferred to avoid the loss of taxonomic diversity. However, the use of ribosomal gene databases (GreenGenes and RDP) with Kraken2 can complement the classification, although they have limitations compared to PlusPFP owing to differences in the coverage and distribution of taxa in the metagenomes. These discrepancies deserve further analysis, and detailed experiments should be performed with combined databases to exhaustively examine bacterial diversity in the soil and rhizosphere (3, 4).

1. Wood, D.E., Lu, J. & Langmead, B. Improved metagenomic analysis with Kraken 2. *Genome Biol* **20**, 257 (2019). https://doi.org/10.1186/s13059-019-1891-0

2. Lu J, Rincon N, Wood DE, Breitwieser FP, Pockrandt C, Langmead B, Salzberg SL, Steinegger M. Metagenome analysis using the Kraken software suite. Nat Protoc. 2022 Dec;17(12):2815-2839. doi: 10.1038/s41596-022-00738-y.
3. Edwin NR, Fitzpatrick AH, Brennan F, Abram F, O'Sullivan O. An in-depth evaluation of metagenomic classifiers for soil microbiomes. Environ Microbiome. 2024 Mar 28;19(1):19. doi: 10.1186/s40793-024-00561-w.
4. Wright RJ, Comeau AM, Langille MGI. From defaults to databases: parameter and database choice dramatically impact the performance of metagenomic taxonomic classification tools. Microb Genom. 2023 Mar;9(3):000949. doi: 10.1099/mgen.0.000949. PMID: 36867161; PMCID: PMC10132073
5. Lu J, Salzberg SL. Ultrafast and accurate 16S rRNA microbial community analysis using Kraken 2. Microbiome. 2020 Aug 28;8(1):124. doi: 10.1186/s40168-020-00900-2. PMID: 32859275; PMCID: PMC7455996.

Rarefaction analysis.

The sequencing depth was substantial for a complex community, such as the soil and rhizosphere (approximately 12-14 Gb; S4 Table; S10 Figure). We conducted a comparison of the sequencing depth for rhizosphere and bulk soil in other cultivated plants, including tomato, cucumber, maize, wheat, beans (other study), and the model plant *Arabidopsis*, with our metagenomic samples (S9A Fig). The number of sequenced metagenomic reads in these plants was significantly lower than that in our study (six–eight times less); however, the calculated species richness was nearly equivalent. Upon normalizing all the sequence reads of the metagenomes studied here to the same number of sequences reads of the other plants, we observed an asymptote in all metagenomes, indicating adequate coverage of diversity (S9B Fig). Given that we collected an average of 80 million reads in average, we determined that normalization of the data was unnecessary.

Compositional structure of the metagenomes.

To address the compositional structure of the metagenomes, we first transformed the read count in the metagenomes using centered log-ratio transformation (CLR) to compute abundance changes without bias in the relative abundance measure. Using 1472 taxa at the genus level (classified by Kraken2), we determined that 80 out of 1472 samples had zero-percentage less than 0.1%. Then, using the CLR matrix, we performed PCoA analysis with the Aitchinson distance matrix to examine the differences between bulk soil and rhizosphere bacterial community abundance and between pairs of rhizosphere communities of the three cultivars (Pinto Saltillo, Bayo, and Black cultivar; S10 Fig.). Rhizosphere communities significantly differed from soil communities, as indicated by PERMANOVA (S8 Table); $p=0.001$; $F=8.1$; $R^2=0.21$), whereas the Pinto Saltillo rhizosphere communities differed from the bacterial rhizosphere of Bayo ($p=0.25$) and black bean cultivars ($P=0.21$; $F=2.4$; $R^2=0.32$). Furthermore, the rhizosphere communities of Bayo and Black beans did not differ significantly from each other (S8 Table). Similar conclusions were drawn from the data analyzed using the relative abundance data described in the manuscript. Hence, the compositional approach strengthened the previous DESeq2 analysis results.

Another concern of our study was the differential abundance estimated using DESeq2. As discussed above, the metagenomic dataset included all Kraken2 classified taxa. We then obtained a broad spectrum of diversity, as was our objective, but at the cost of including low-abundance, rare, and false-positive taxa. Consequently, this may result in data overdispersion. However, in the metagenome studied, beta-dispersion was reasonably low and did not affect PERMANOVAS when comparing Pinto Saltillo rhizosphere and bulk soil communities (S12 and S13 Figs.). Therefore, we considered that DEseq2 could manage the comparisons of differential abundances shown in Figures 1B and 4B without the need for data normalization. Indeed, the relatively low zero counts in the metagenomes allowed us to use the DESeq2.

We performed ANCOMBC with the CLR abundance matrix to account for the compositional structure in the differential abundance estimates. The assessment of the change in abundance in taxa (at genus level) from soil to the rhizosphere in the three common bean cultivars with ANCOMBC indicated that abundant taxa, upper than two log-fold change (2-LFC) were included in the DESeq2 group of similar LFC (S14 and S15 Figs.). These results indicated that, although ANCONBC is more conservative and identifies less abundant taxa than DESeq2, the conclusions in the manuscript remain valid.
