# Supplementary material for: The rhizosphere of Phaseolus vulgaris L. cultivars hosts a similar bacterial community in local agricultural soils
Source: PLoS One. 2025 Mar 20;20(3):e0319172. doi: 10.1371/journal.pone.0319172 (PMC11925306; doi:10.1371/journal.pone.0319172)
Supplement: S1 Fig — (A) sampling area within the INIFAP Experimental Field, in the state of Zacatecas, México (see geographic coordinates in S1 Table). Plots with agricultural and non-agricultural soil are indicated in the scheme. Nine additional bean cultivars were collected 1.9 km away from the sampling area geographic coordinates in S1 Table. (B) Experimental design of the sampling grid and location of collection sites. Soil and rhizosphere samples were taken at each intersection (yellow dots). Rhizosphere samples consisted of a rhizosphere pool of the soil root of 9 plants. The physicochemical analysis was performed with samples obtained at sites marked by blue dots. Physicochemical measures and soil classification was performed at INIFAP-Zacatecas. (PDF) [file pone.0319172.s002.pdf]

A

B

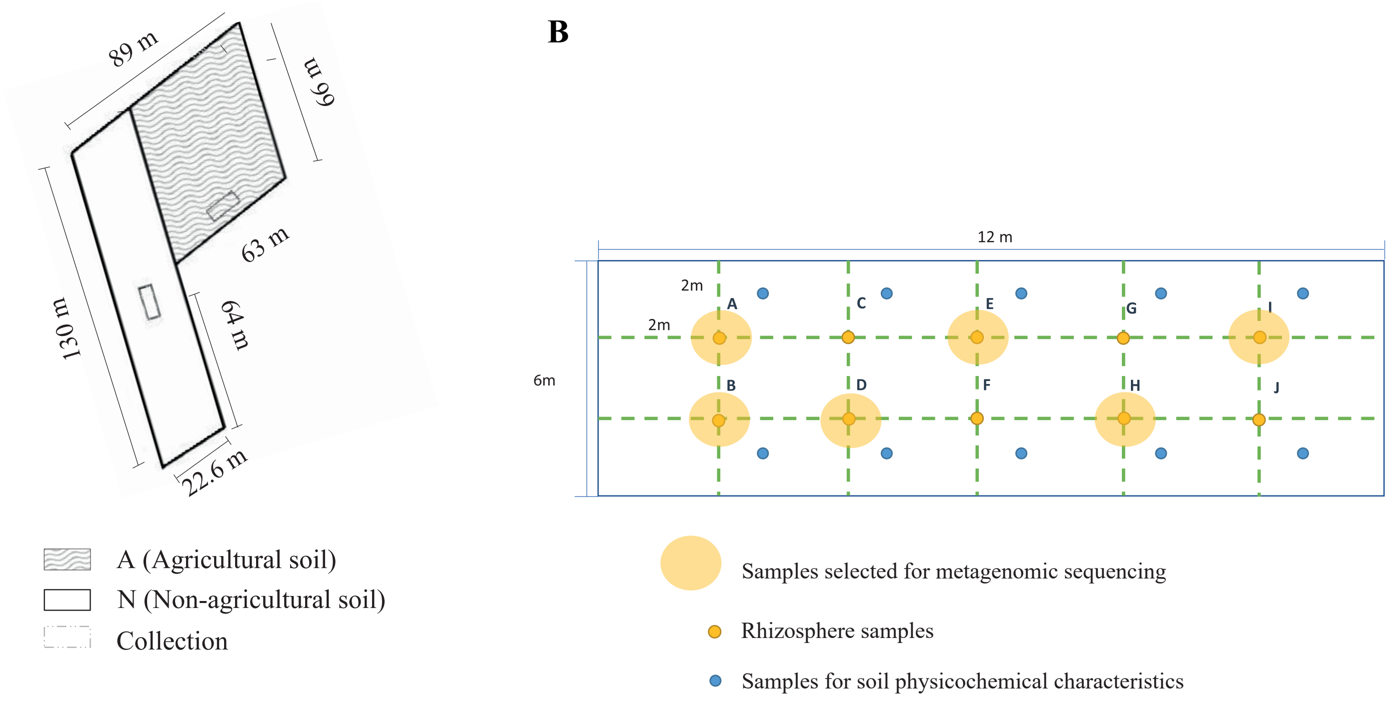

S1 Fig. Location of the sampling site. (A) sampling area within the INIFAP Experimental Field, in the state of Zacatecas, México (see geographic coordinates in S1 Table). Plots with agricultural and non-agricultural soil are indicated in the scheme. Nine additional bean cultivars were collected 1.9 km away from the sampling area geographic coordinates in S1 Table). (B) Experimental design of the sampling grid and location of collection sites. Soil and rhizosphere samples were taken at each intersection (yellow dots). Rhizosphere samples consisted of a rhizosphere pool of the soil root of 9 plants. The physicochemical and soil type analysis was performed at INIFAP-Zacatecas (blue dots).
