# Supplementary material for: The rhizosphere of Phaseolus vulgaris L. cultivars hosts a similar bacterial community in local agricultural soils
Source: PLoS One. 2025 Mar 20;20(3):e0319172. doi: 10.1371/journal.pone.0319172 (PMC11925306; doi:10.1371/journal.pone.0319172)
Supplement: S2 Fig — Principal Component Analysis (PCA) of the soil chemical (pH; Nitrogen, N; Sodium, Na; Magnesium, Mg; Potassium, K; and Phosphorus, P) and physical (Organic Material, OM; Field Capacity, FC; Saturation Point, SP; Permanent Wilting Point, PWP; Electric Conductivity, EC) properties. PERMANOVA statistic with Euclidian distance is shown below the plot. (PDF) [file pone.0319172.s003.pdf]

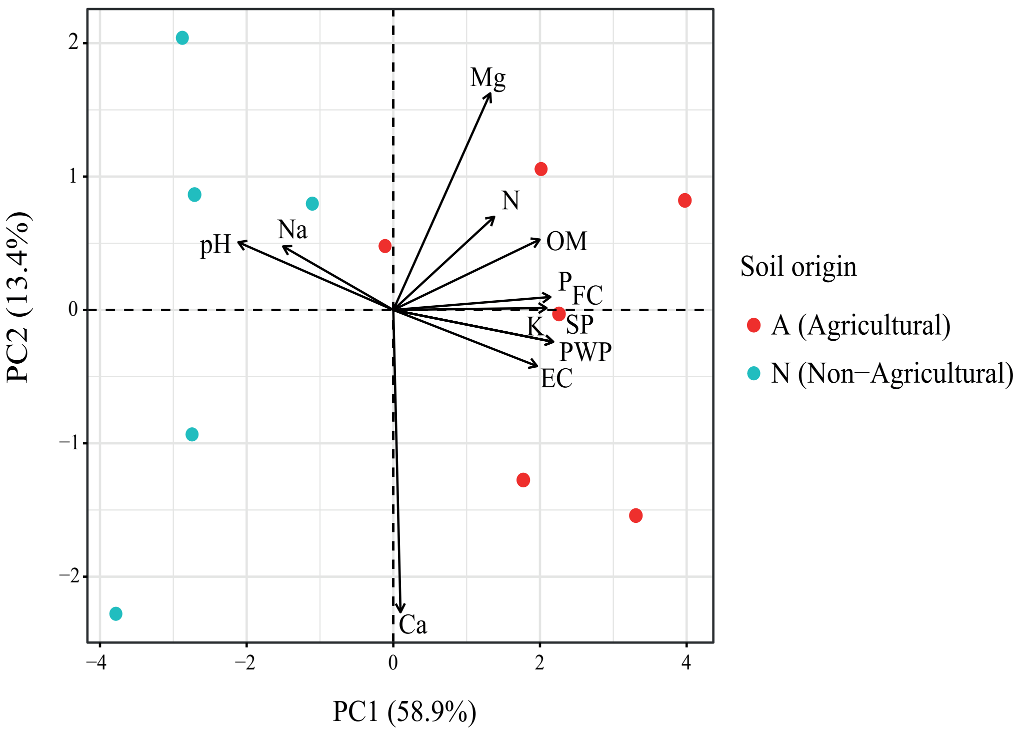

SumOfSqs=958.76, R2=0.64161, F=16.112, pval=0.002
Residual SumOfSqs=535.55, Residual R2=0.35839

S2 Fig. Physicochemical characteristics of agriculture (A) and non-agriculture (N) soil. Principal Component Analysis (PCA) of the soil chemical (pH, N, Mg, K, and P) and physical (OM, FC, SP, PWP, and EC) properties. PERMANOVA statistic with Euclidian distance is shown.
