# Supplementary material for: The rhizosphere of Phaseolus vulgaris L. cultivars hosts a similar bacterial community in local agricultural soils
Source: PLoS One. 2025 Mar 20;20(3):e0319172. doi: 10.1371/journal.pone.0319172 (PMC11925306; doi:10.1371/journal.pone.0319172)
Supplement: S3 Fig — Sequence reads assigned to virus are also included. Kraken2 was used with the default parameters (confidence 0.0, minimum-hit-groups 2). (PDF) [file pone.0319172.s004.pdf]

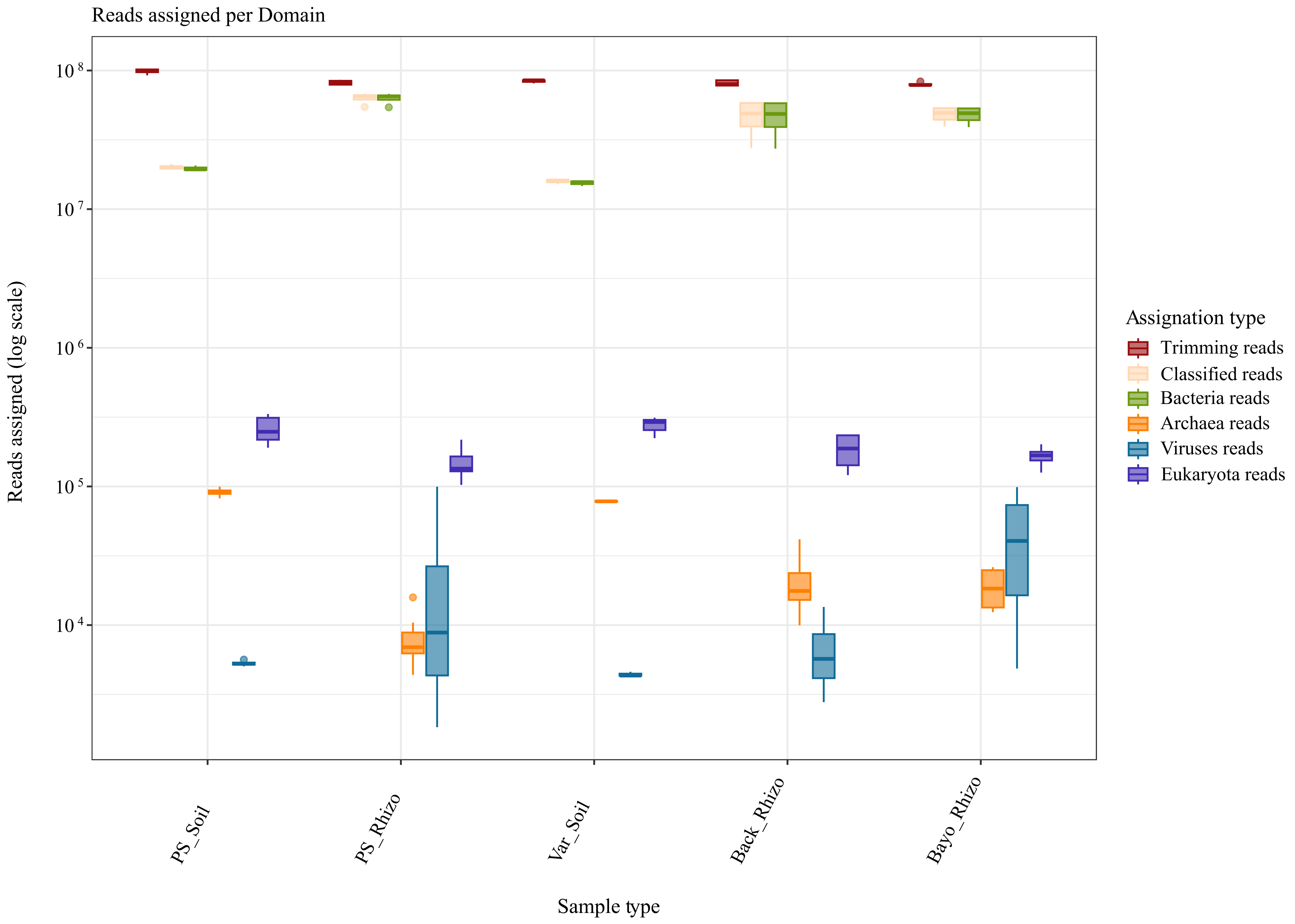

S3 Fig. Classified sequence reads by Kraken2 whith default parameters (confidence 0.0, minimum-hit-groups 2).
