# Supplementary material for: The rhizosphere of Phaseolus vulgaris L. cultivars hosts a similar bacterial community in local agricultural soils
Source: PLoS One. 2025 Mar 20;20(3):e0319172. doi: 10.1371/journal.pone.0319172 (PMC11925306; doi:10.1371/journal.pone.0319172)
Supplement: S4 Fig — Metagenomic sequences came from bulk soil and rhizosphere of agriculture (A) and non-agriculture (N) soils of Pinto Saltillo common bean. (PDF) [file pone.0319172.s005.pdf]

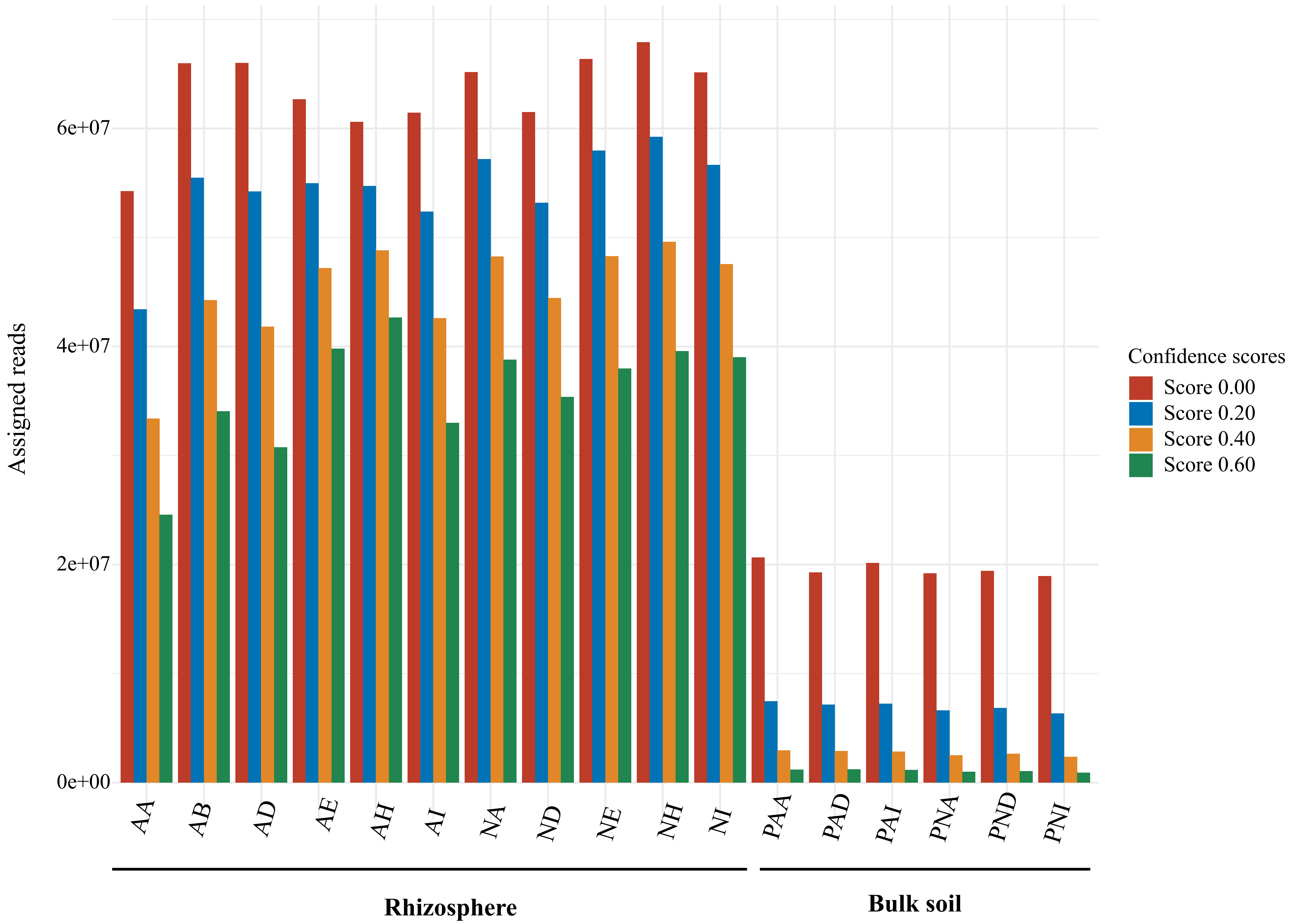

S4 Fig. Classified sequence reads from rhizosphere and bulk soil metagenomes at different confidence scores. Metagenomic sequences came from bulk soil and rhizosphere of agriculture (A) and non-agriculture (N) of Pinto Saltillo common bean.
