# Supplementary material for: The rhizosphere of Phaseolus vulgaris L. cultivars hosts a similar bacterial community in local agricultural soils
Source: PLoS One. 2025 Mar 20;20(3):e0319172. doi: 10.1371/journal.pone.0319172 (PMC11925306; doi:10.1371/journal.pone.0319172)
Supplement: S6 Fig — Shannon index was estimated with the taxonomic classification of Kraken 2 at different confidence scores (inset). T-test statistics between samples is shown. (PDF) [file pone.0319172.s007.pdf]

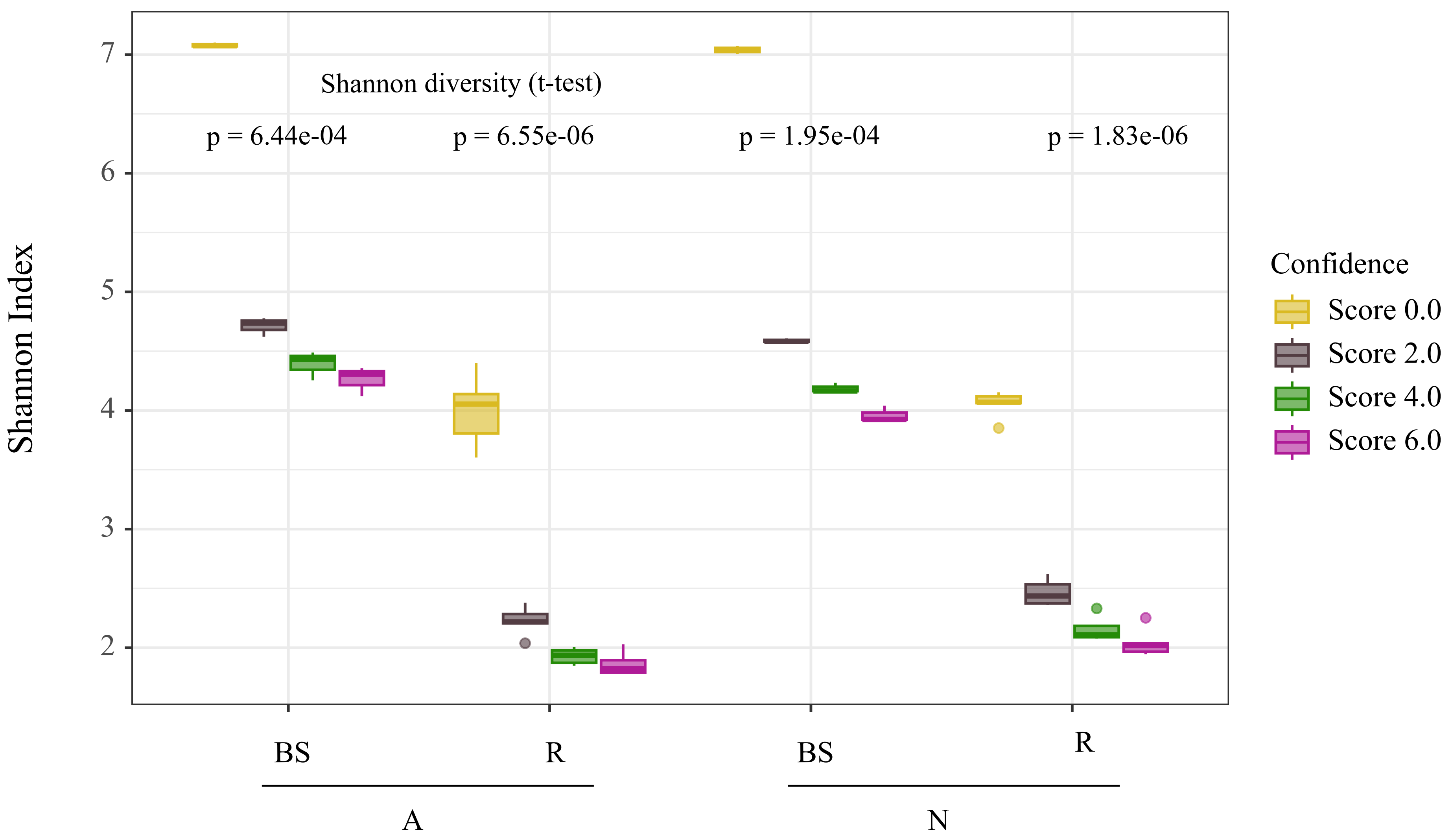

S6 Fig. Shannon diversity index of bacteria communities in Bulk Soil (BS) and Rhizosphere of common bean Pinto Saltillo (R). Shannon index was estimated with the taxonomic classification of Kraken2 at different cofidence scores.
