# Supplementary material for: The rhizosphere of Phaseolus vulgaris L. cultivars hosts a similar bacterial community in local agricultural soils
Source: PLoS One. 2025 Mar 20;20(3):e0319172. doi: 10.1371/journal.pone.0319172 (PMC11925306; doi:10.1371/journal.pone.0319172)
Supplement: S7 Fig — (A) Comparison of the number of taxonomic units at genus level determined with Kraken2 using the ribosomal data bases RDP, GreenGenes, and Plus-PFP. (B) Number and percent of shared genera between Kraken2 classifications with the PlusPFP (KRAKEN2) and ribosomal data bases (GreenGenes, GREENG; and RDP, RDP_DTB). (PDF) [file pone.0319172.s008.pdf]

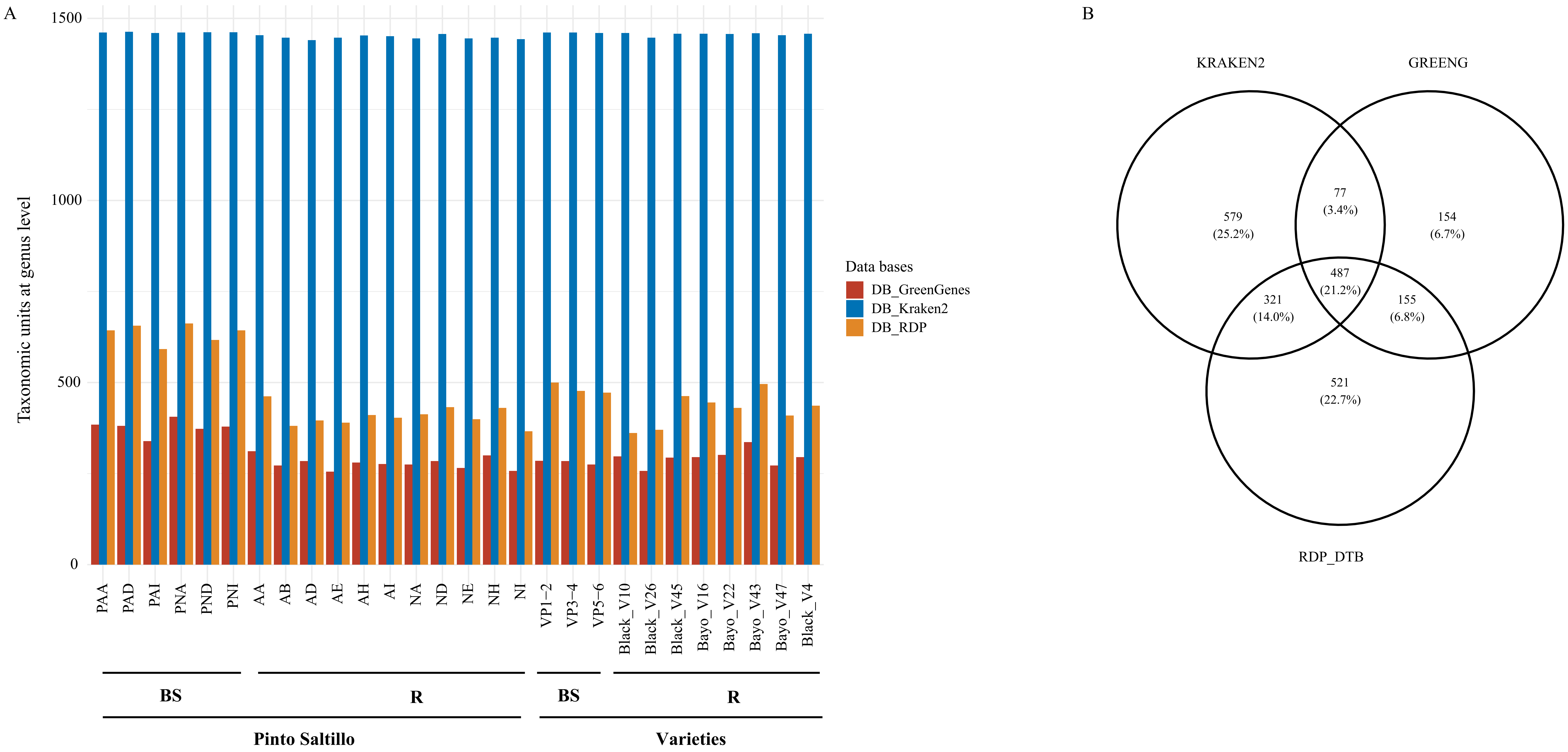

S7 Fig . Comparison of the number of taxonomic units at genus level determined with Kraken2 using the ribosomal data bases RDP, GreenGenes, and Plus-PFP. Number and percent of shared genera between Kraken2 classifications with the PlusPFP and ribosomal data bases (GreenGenes and RDP).
