# Supplementary material for: The rhizosphere of Phaseolus vulgaris L. cultivars hosts a similar bacterial community in local agricultural soils
Source: PLoS One. 2025 Mar 20;20(3):e0319172. doi: 10.1371/journal.pone.0319172 (PMC11925306; doi:10.1371/journal.pone.0319172)
Supplement: S8 Fig — A. PlusPFP (n = 1464) B. RDP (n = 1484.) Most of the classified genus in A (95%) were shared in the metagenomes. (PDF) [file pone.0319172.s009.pdf]

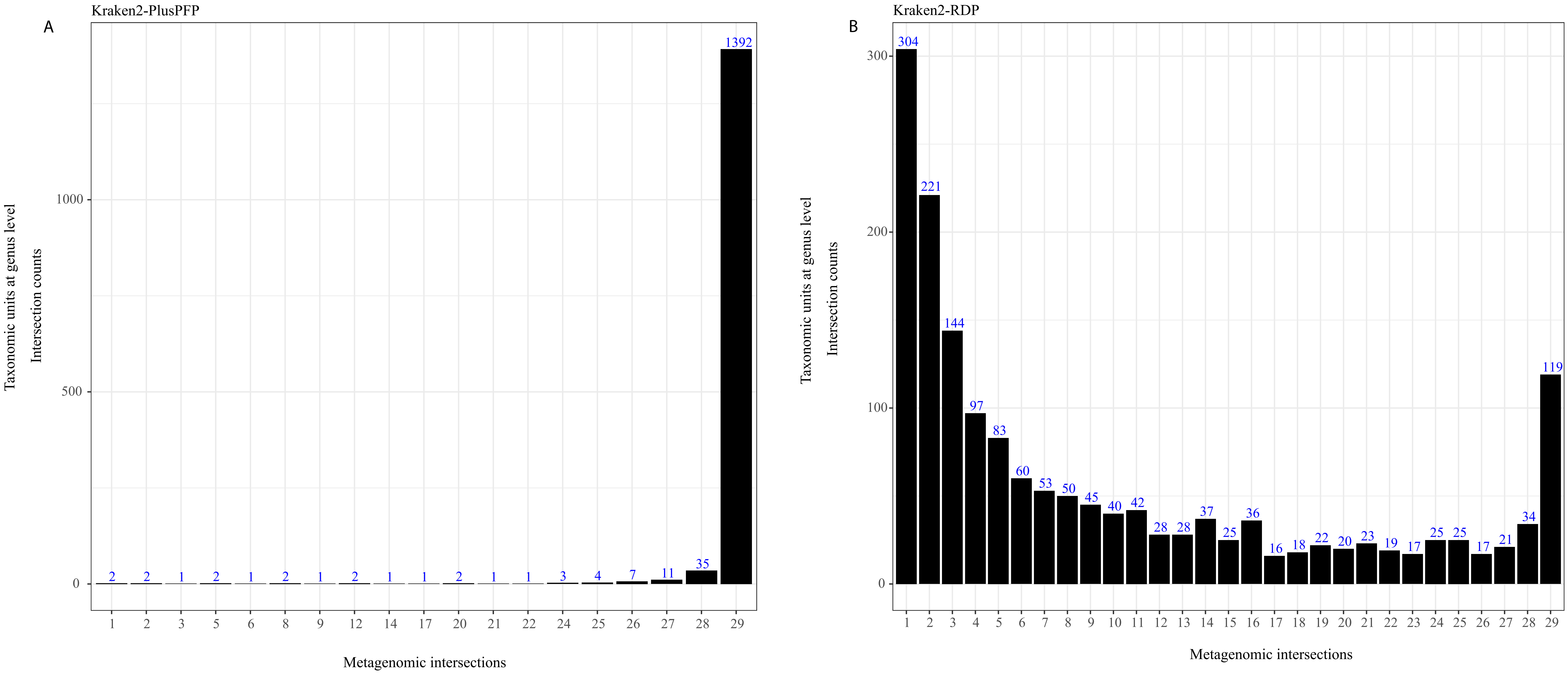

S8 Fig. U-Plots of the distribution of bacterial genus classified with Kraken2 using the data bases: A. PlusPFP (n=1464) B. RDP (n= 1484.) Most of the classified genus in A (95%) were shared in the metagenomes.
