# Supplementary material for: The rhizosphere of Phaseolus vulgaris L. cultivars hosts a similar bacterial community in local agricultural soils
Source: PLoS One. 2025 Mar 20;20(3):e0319172. doi: 10.1371/journal.pone.0319172 (PMC11925306; doi:10.1371/journal.pone.0319172)
Supplement: S9 Fig — Sample size is the number of metagenomic sequence reads in the rarefaction statistics. Species richness is the number of species classified by Kraken2. Green rectangle corresponded to the rarefaction of metagenomic sequences of bulk soil and rhizosphere of common bean in this study. A. All metagenomics samples without adjustment to the same sample size. B. All metagenomic sequences with the samples normalized to 20 million reads. Metagenomes Arabidopsis, bean-Mendes, cucumber, maize, tomato, and wheat raw metagenomic sequence were downloaded from GenBank (NCBI) and processed with the same procedures described in this work (see methods). (PDF) [file pone.0319172.s010.pdf]

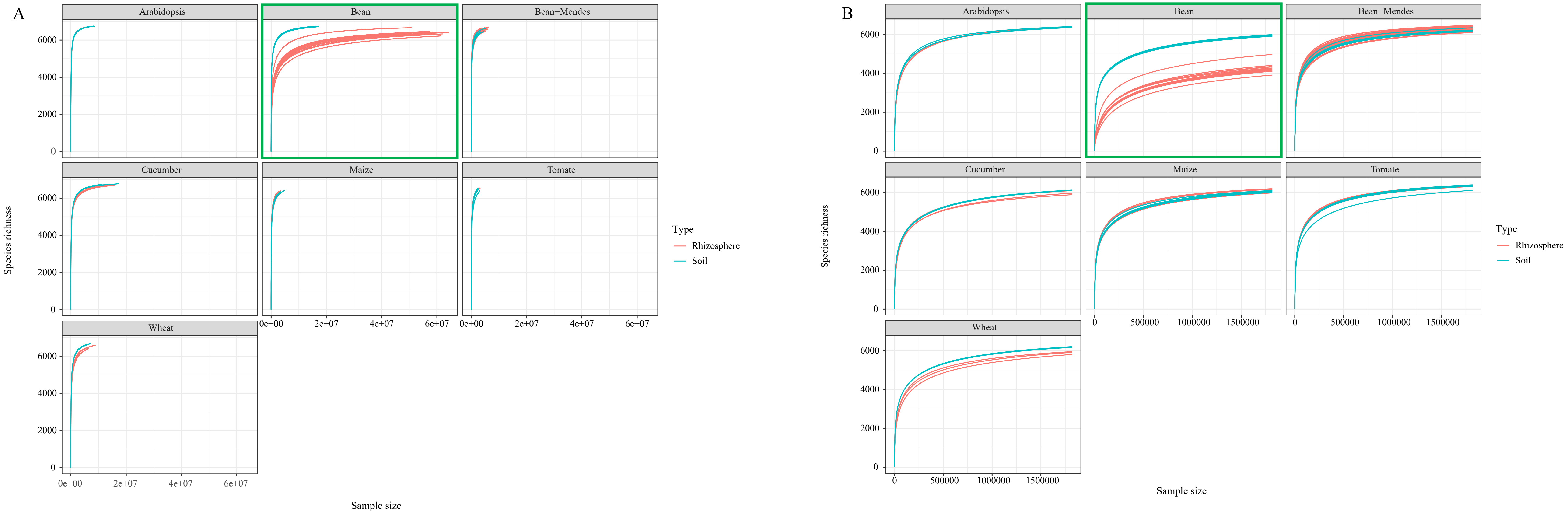

S9 Fig. Rarefaction curves of bulk soil and rhizosphere metagenomes of distinct cultivated plants and common Bean (this study). Sample size is the number of metagenomic sequence reads employeed in the rarefaction statistics. Species richnes is the number of species classified by Kraken2. Green rectangle corresponded to the rarefaction of metagenomic sequences of bulk soil and rhizosphere of common bean in this study. A. All metagenomics samples without adjustement to the same sample size. B. All metagenomic sequences with the samples normalised to 20 million reads. Metagenomes of Arabidopsis, bean-Mendes, cucumber, maize, tomato, and wheat raw metagenomic sequence were dowloaded from GenBank (NCBI) and processed with the same procedures described in this work (see methods).
