# Supplementary material for: The rhizosphere of Phaseolus vulgaris L. cultivars hosts a similar bacterial community in local agricultural soils
Source: PLoS One. 2025 Mar 20;20(3):e0319172. doi: 10.1371/journal.pone.0319172 (PMC11925306; doi:10.1371/journal.pone.0319172)
Supplement: S10 Fig — Abundance matrix was calculated with the CLR approach and PCoA with the Aitchinson distances. Statistical analysis with PERMANOVA is shown in S8 Table. (PDF) [file pone.0319172.s011.pdf]

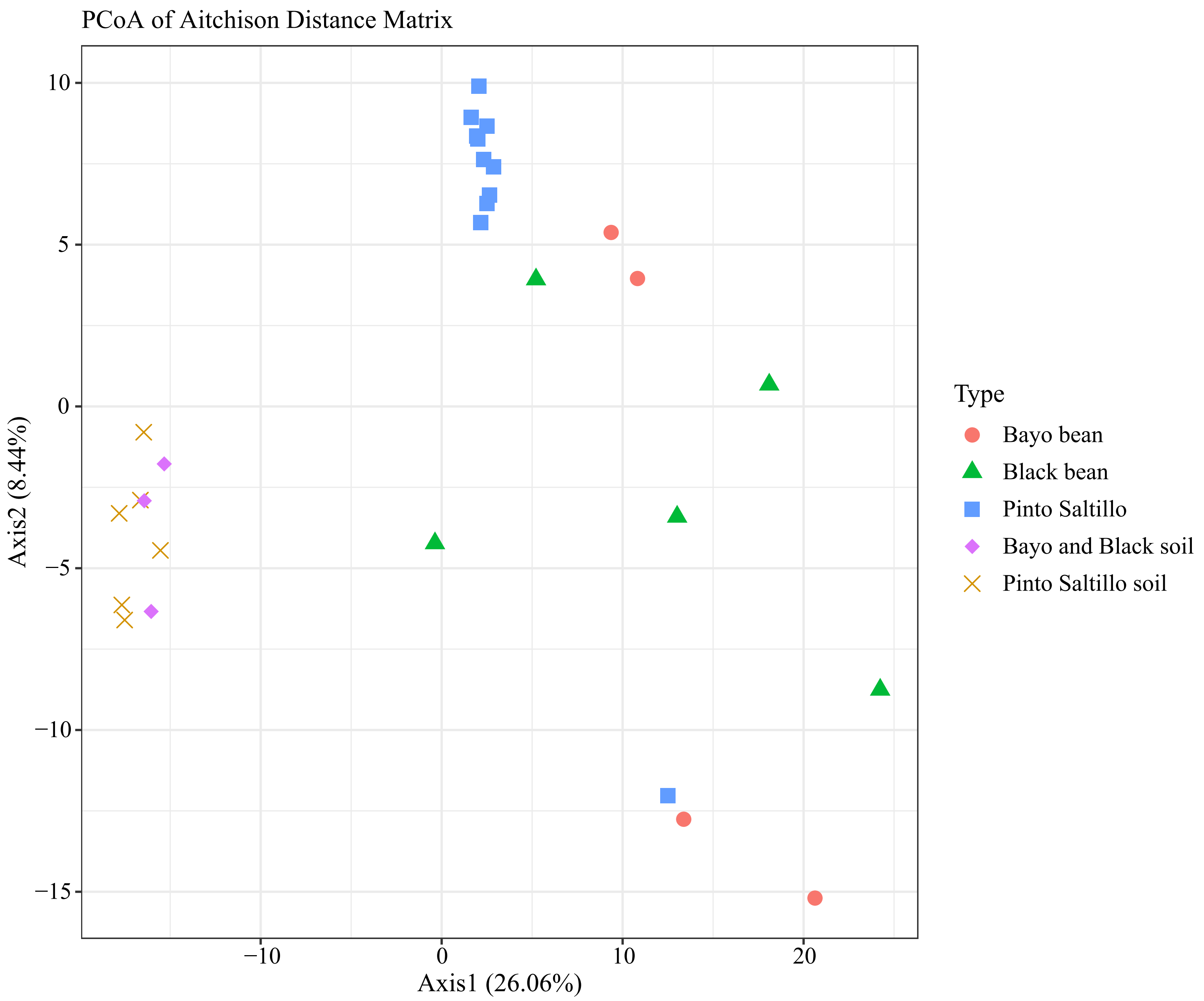

S10 Fig. Beta diversity of bacterial communities of common bean cultivars (Pinto Saltillo, Black, and Bayo), from soil and rhizosphere. Abundance matrix was calculated with the CLR approach and pCoA with the Aitchinson distances. Statistic analysis with PERMANOVA are shown in S8 Table.
