# Supplementary material for: The rhizosphere of Phaseolus vulgaris L. cultivars hosts a similar bacterial community in local agricultural soils
Source: PLoS One. 2025 Mar 20;20(3):e0319172. doi: 10.1371/journal.pone.0319172 (PMC11925306; doi:10.1371/journal.pone.0319172)
Supplement: S11 Fig — A. Venn diagram of shared genera classified by Kraken2 and BRACKEN (using the PlusPFP), and Kraken2 using the RPD. B. Beta diversity of bacterial communities of the common bean cultivar Pinto Saltillo, from bulk soil and rhizosphere. Abundance matrix was calculated with BRACKEN. PCoA shows Bray Curtis distances between samples. (PDF) [file pone.0319172.s012.pdf]

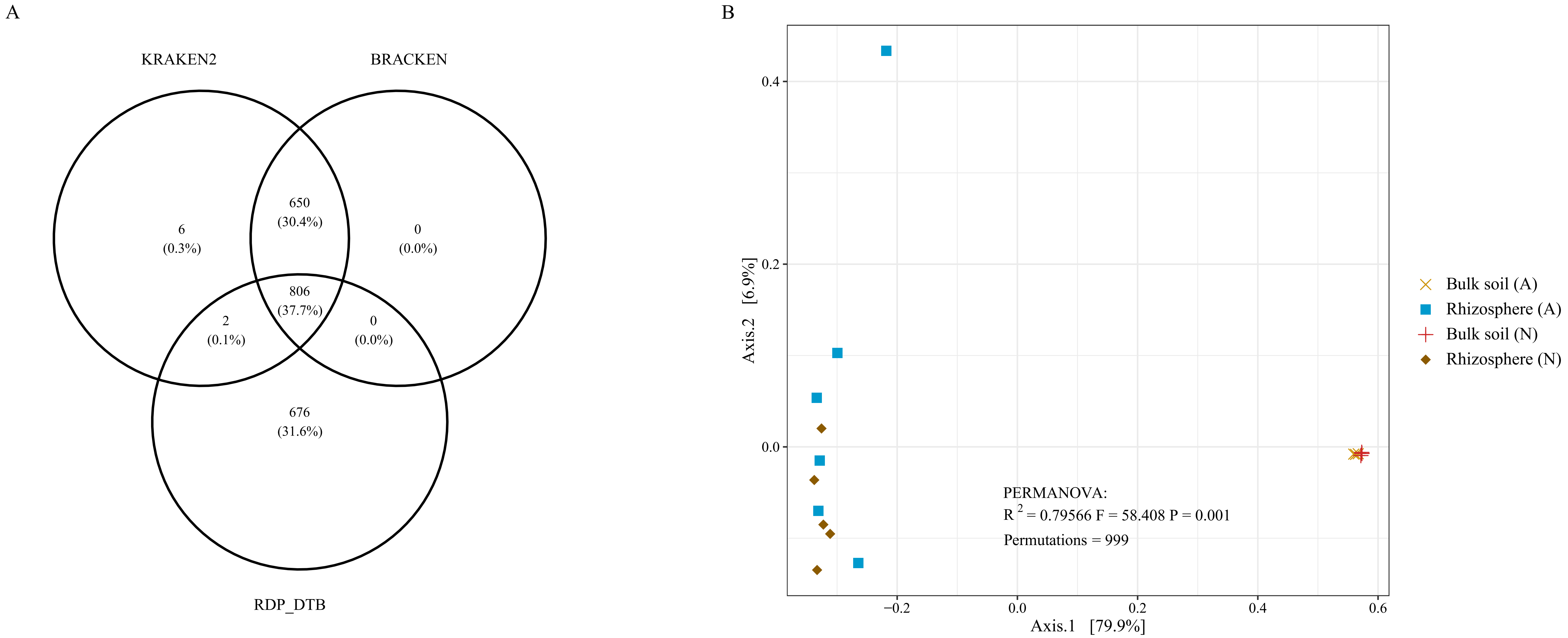


S11 Fig. A. Comparisons of genus classified by Kraken2 and BRACKEN (using the PlusPFP), and Kraken2 using the RPD. B. Beta diversity of bacterial communities of the common bean cultivar Pinto Saltillo, from bulk soil and rhizosphere. Abundance matrix was calculated with BRACKEN. pCoA shows Bray Curtis distances between samples.
