# Supplementary material for: The rhizosphere of Phaseolus vulgaris L. cultivars hosts a similar bacterial community in local agricultural soils
Source: PLoS One. 2025 Mar 20;20(3):e0319172. doi: 10.1371/journal.pone.0319172 (PMC11925306; doi:10.1371/journal.pone.0319172)
Supplement: S12 Fig — With the outlier sample AH (A, top left) and without the outliers (B). Beta dispersion distance to the centroid is shown in plots C and D. Statistical Levene´s test is shown in the table E. (PDF) [file pone.0319172.s013.pdf]

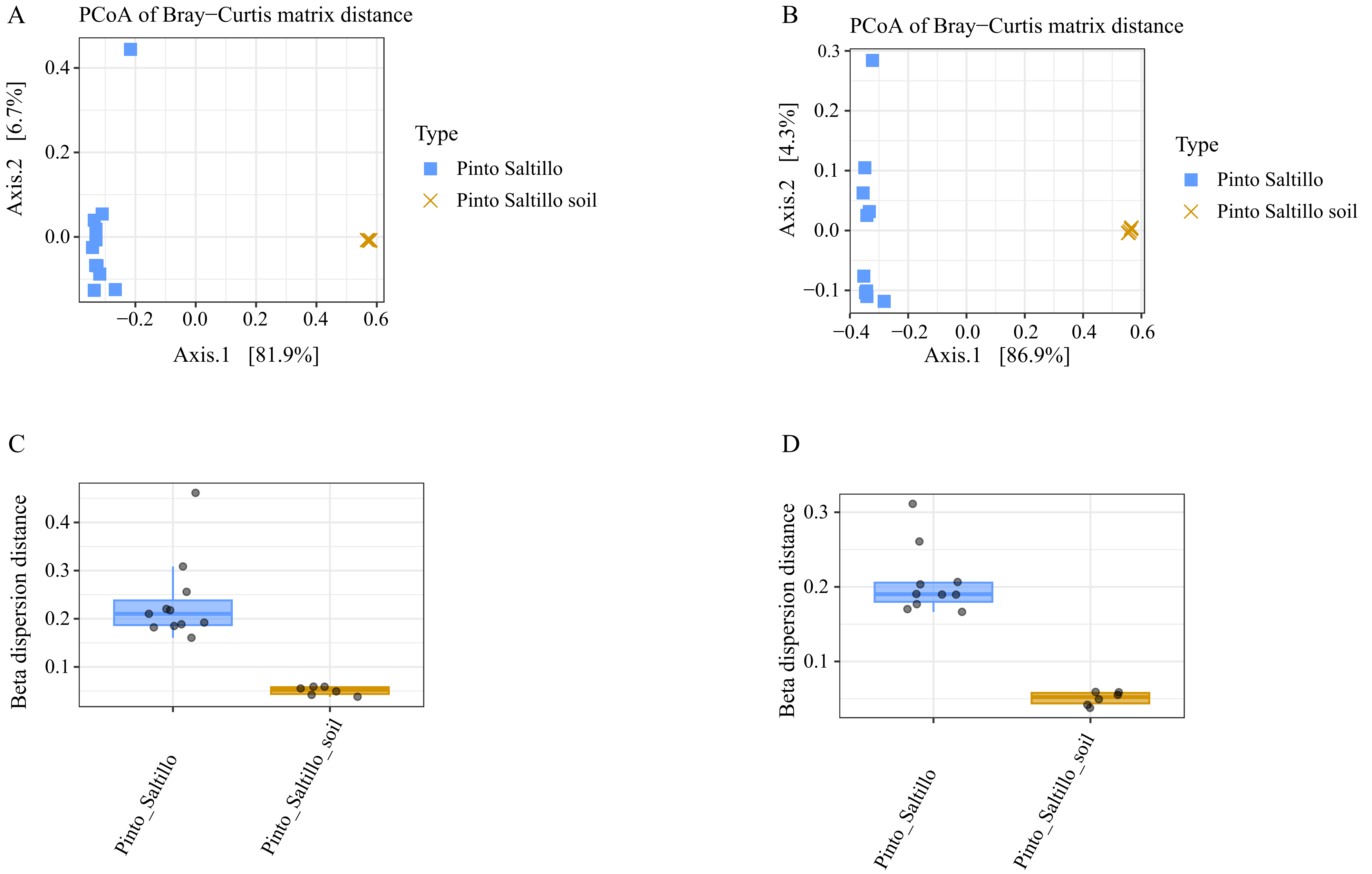

E

Beta dispersion of Bray Curtis matrix distance (samples without transformation/normalization)											
Dispersion Pinto Saltillo samples and Soil Pinto Saltillo Samples (without AH)											
#Average distance to centroid:											
Pinto_Saltillo	Soil_Pinto_Saltillo					Levene's Test for Homogeneity of Variance (center = median)					
0.20652	0.05039						Df	F value	Pr(>F)		
						Group	1	1.6601	0.2185		
						Residual	14				
Dispersion Pinto Saltillo samples and Soil Pinto Saltillo Samples (all sample)											
#Average distance to centroid:											
Pinto_Saltillo	Soil_Pinto_Saltillo					Levene's Test for Homogeneity of Variance (center = median)					
0.23476	0.05039						Df	F value	Pr(>F)		
						Group	1	2.1044	0.1675		
						Residual	15				

S12 Fig. Beta dispersion analysis of bulk soil and rhizosphere samples of Pinto Saltillo cultivar with the outlier sample AH (A, top left) and without the outlier (B). Beta dispersion distance to the centroid is shown in plots C and D. Statistical Levene´s test are shown in the table E.
