# Supplementary material for: The rhizosphere of Phaseolus vulgaris L. cultivars hosts a similar bacterial community in local agricultural soils
Source: PLoS One. 2025 Mar 20;20(3):e0319172. doi: 10.1371/journal.pone.0319172 (PMC11925306; doi:10.1371/journal.pone.0319172)
Supplement: S13 Fig — Levene´s test of homogeneity is shown below the figure. (PDF) [file pone.0319172.s014.pdf]

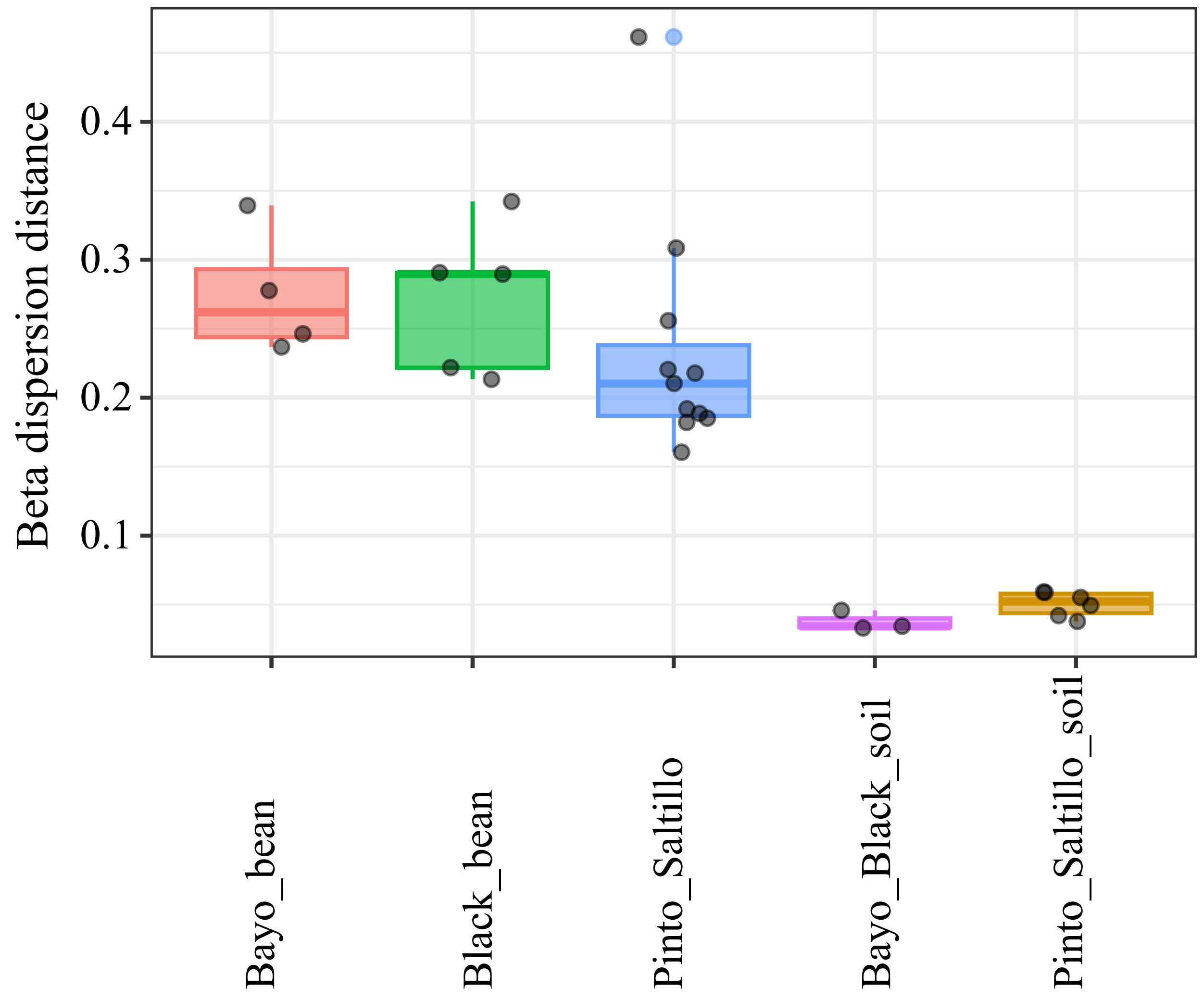

Dispersion All Samples				
#Average distance to centroid:				
Pinto_Saltillo	Soil_Pinto_Saltillo	Negro	Bayo	Soil_other_varieties
0.23476	0.05039	0.27153	0.27507	0.03775

Levene's Test for Homogeneity of Variance (center = median)			
	Df	F value	Pr(>F)
Group	4	1.007	0.4234
Residual	24		

S13 Fig. Beta dispersion test of the metagenomic samples from bulk soil and rhizosphere of Bayo, Black, and Pinto Saltillo cultivars. Levene´s test is shown below.
