# Supplementary material for: The rhizosphere of Phaseolus vulgaris L. cultivars hosts a similar bacterial community in local agricultural soils
Source: PLoS One. 2025 Mar 20;20(3):e0319172. doi: 10.1371/journal.pone.0319172 (PMC11925306; doi:10.1371/journal.pone.0319172)
Supplement: S14 Fig — Plots of the rhizosphere bacterial community cultivars A, Black bean. B, Bayo bean, and C, Pinto Saltillo. They were compared with the respective bulk soil bacterial community. (PDF) [file pone.0319172.s015.pdf]

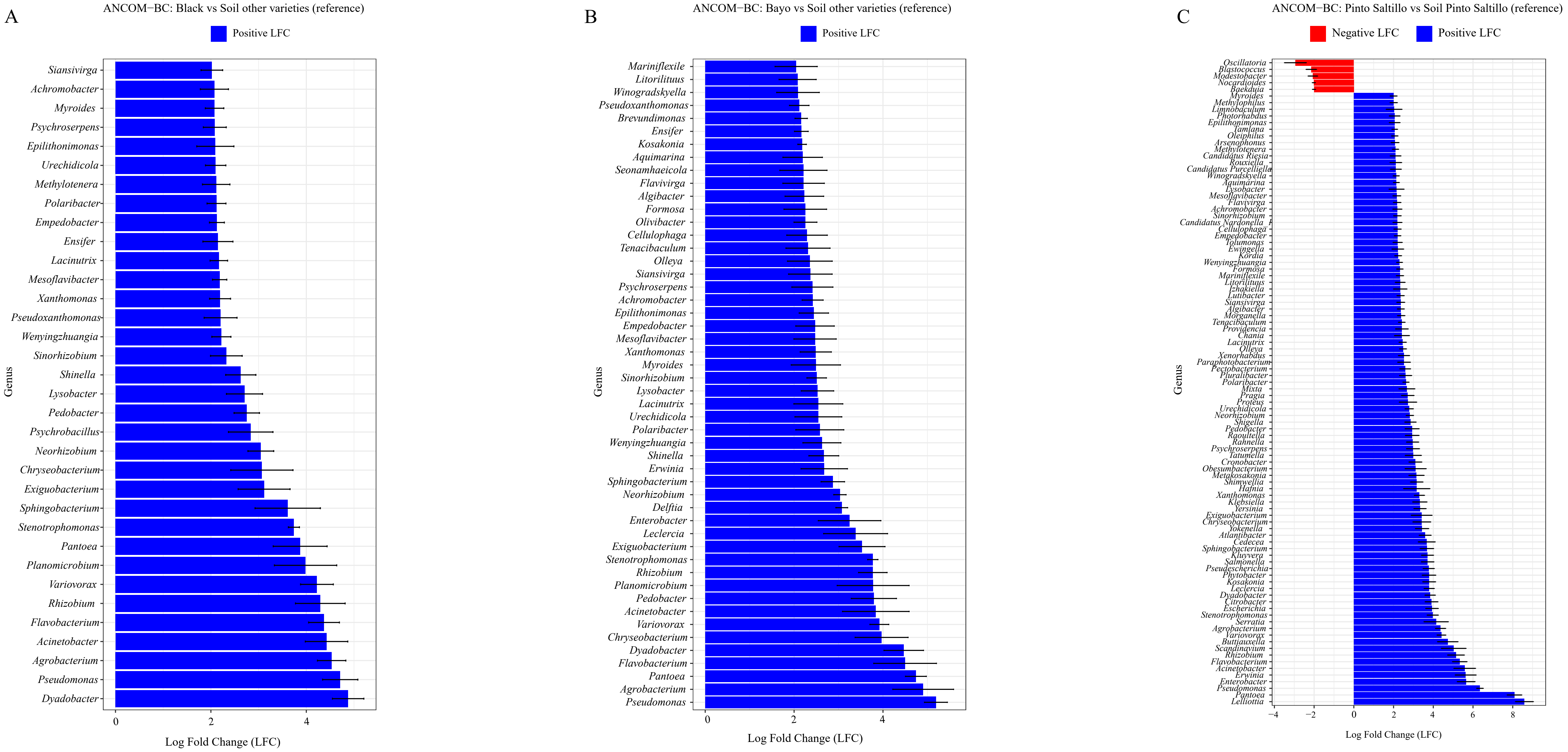


S14 Fig.. ANCOM differential abundance plots of the rhizosphere bacterial community cultivars A. Black bean., B. Bayo bean, and C. Pinto Saltillo. They were compared with the respective bulk soil bacterial community.
