# Supplementary material for: The rhizosphere of Phaseolus vulgaris L. cultivars hosts a similar bacterial community in local agricultural soils
Source: PLoS One. 2025 Mar 20;20(3):e0319172. doi: 10.1371/journal.pone.0319172 (PMC11925306; doi:10.1371/journal.pone.0319172)
Supplement: S15 Fig — A, Pinto Saltillo, B, Black bean, C. Bayo bean. (PDF) [file pone.0319172.s016.pdf]

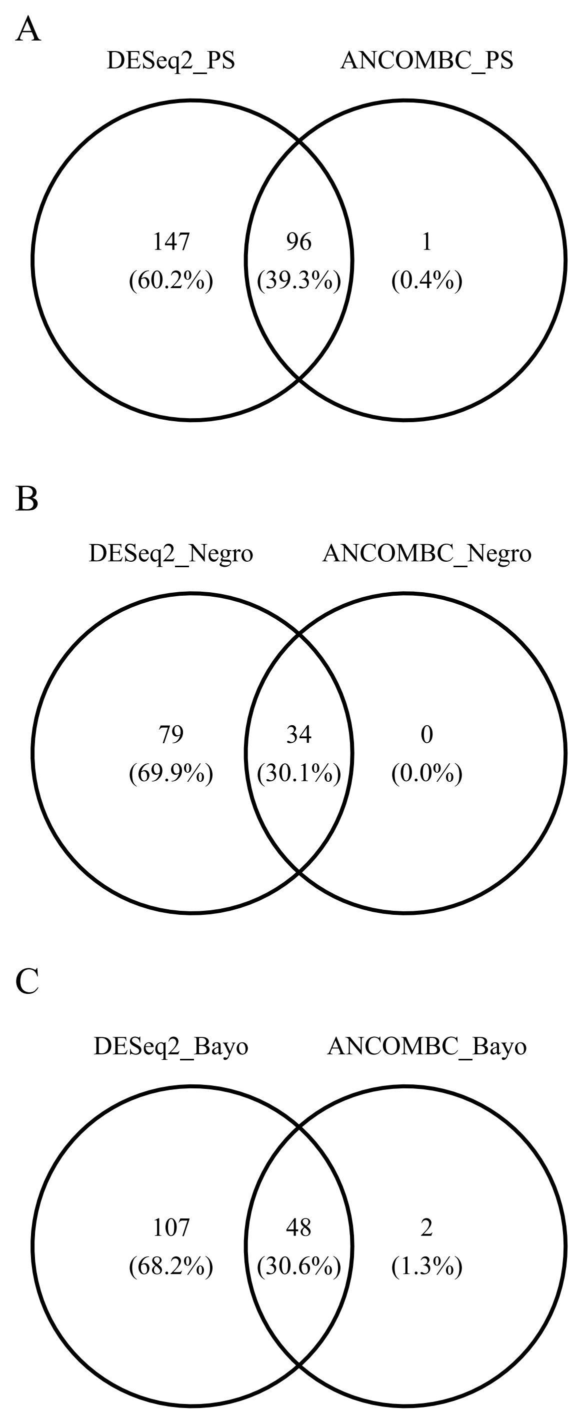

S15 Fig. Common taxa at genus level differentially abundant (two log-fold) in the bacterial rhizosphere community, evaluated with DESeq2 and ANCOMBC. A. Pinto Saltillo, B. Black bean, and C. Bayo bean.
