# Supplementary material for: The rhizosphere of Phaseolus vulgaris L. cultivars hosts a similar bacterial community in local agricultural soils
Source: PLoS One. 2025 Mar 20;20(3):e0319172. doi: 10.1371/journal.pone.0319172 (PMC11925306; doi:10.1371/journal.pone.0319172)
Supplement: S16 Fig — Cultivar Pinto Saltillo grown in Non-agricultural soil (A) and agriculture soil (B). Venn diagram shows the common taxa at species level, determined with Kraken2 (C). (PDF) [file pone.0319172.s017.pdf]

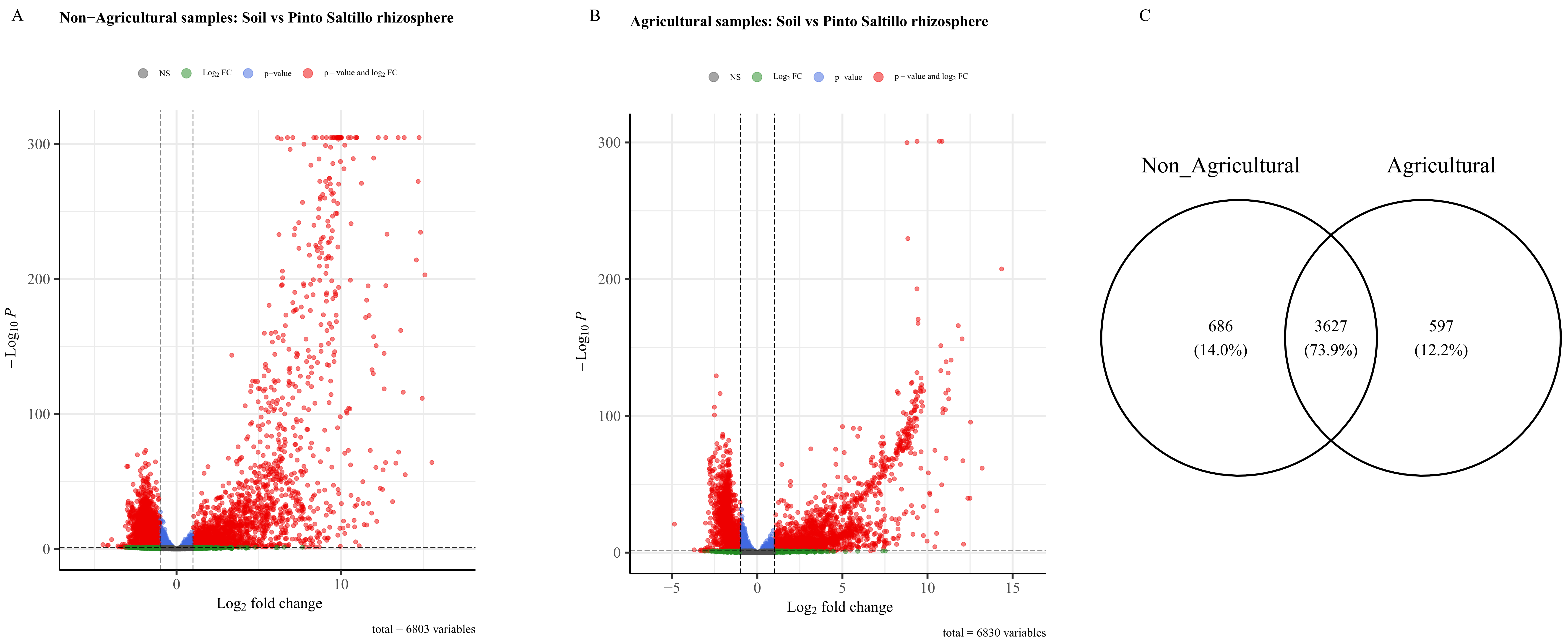

S16 Fig. Differential abundance of the bacterial communities in the rhizosphere of cultivar Pinto Saltillo grown in Non-agricultural soil (A) and agricultural soil (B). Venn diagram shows the common taxa at specieslevel, determined with Kraken2 (C).
