# Supplementary material for: The rhizosphere of Phaseolus vulgaris L. cultivars hosts a similar bacterial community in local agricultural soils
Source: PLoS One. 2025 Mar 20;20(3):e0319172. doi: 10.1371/journal.pone.0319172 (PMC11925306; doi:10.1371/journal.pone.0319172)
Supplement: S17 Fig — The stacked bars display 37 bacterial phyla. In bulk soil, the most prevalent phyla are Actinobacteriota (orange) and Pseudomonadota (green). Conversely, in the rhizosphere, Pseudomonadota comprise over 90% of the community. (PDF) [file pone.0319172.s018.pdf]

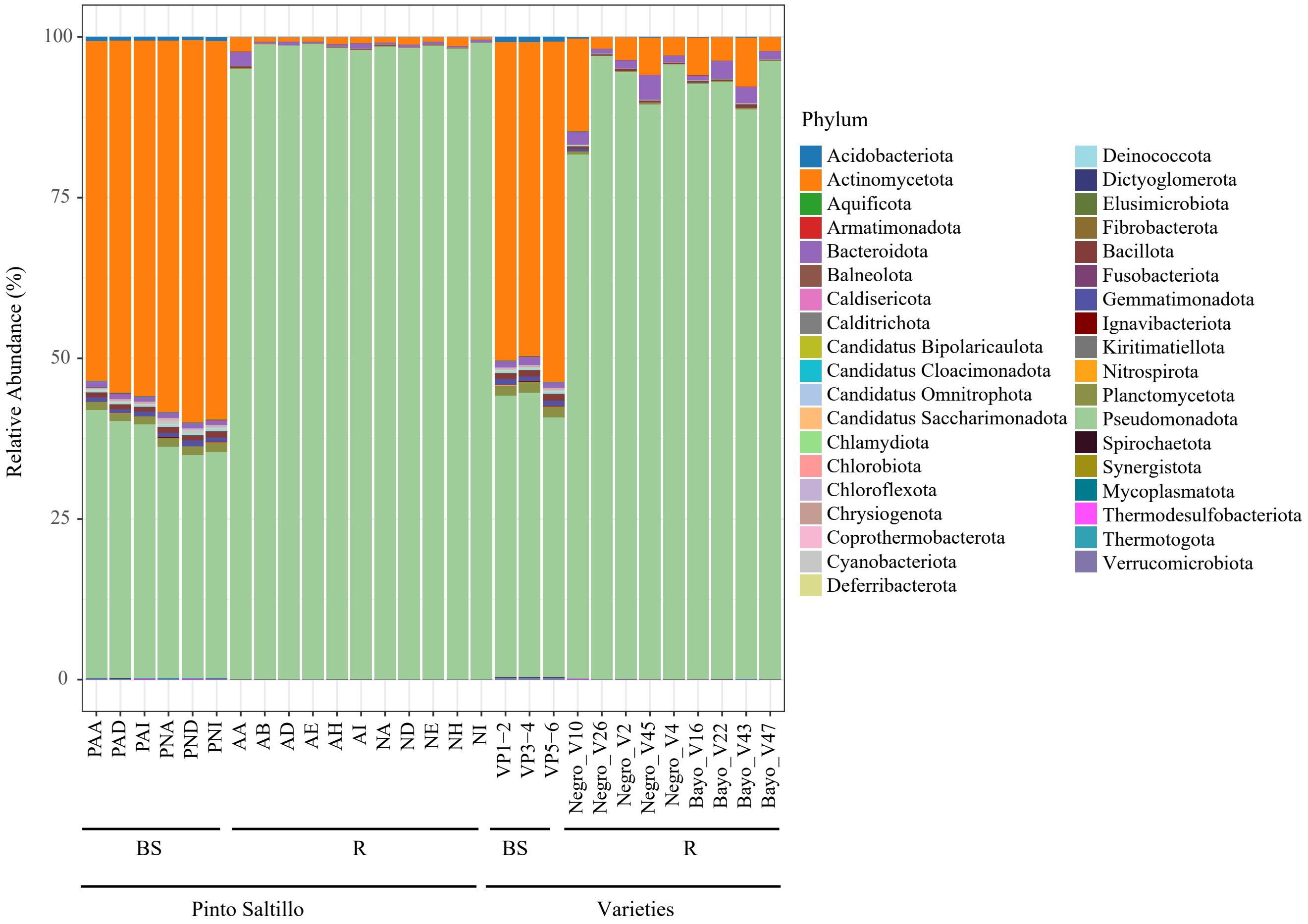

S17 Fig Relative abundance of bacterial phyla presents in bulk soil and rhizosphere. The stacked bars display 37 bacterial phyla. In bulk soil, the most prevalent phyla are Actinobacteriota (orange) and Pseudomonadota (green). Conversely, in the rhizosphere, Proteobacteria phyla comprise over 90% of the community.
