# Supplementary material for: The rhizosphere of Phaseolus vulgaris L. cultivars hosts a similar bacterial community in local agricultural soils
Source: PLoS One. 2025 Mar 20;20(3):e0319172. doi: 10.1371/journal.pone.0319172 (PMC11925306; doi:10.1371/journal.pone.0319172)
Supplement: S18 Fig — The stacked bars indicate the abundance of bacterial classes in bulk soil. Actinobacteria (red), Alpha-Proteobacteria (blue), and Beta-Proteobacteria (orange). Rhizosphere is represented by Gamma-Proteobacteria (light blue) and Alpha-Proteobacteria (blue), and Beta-Proteobacteria (orange). Bacterial classes with less than 1% abundance are shown in gray. (PDF) [file pone.0319172.s019.pdf]

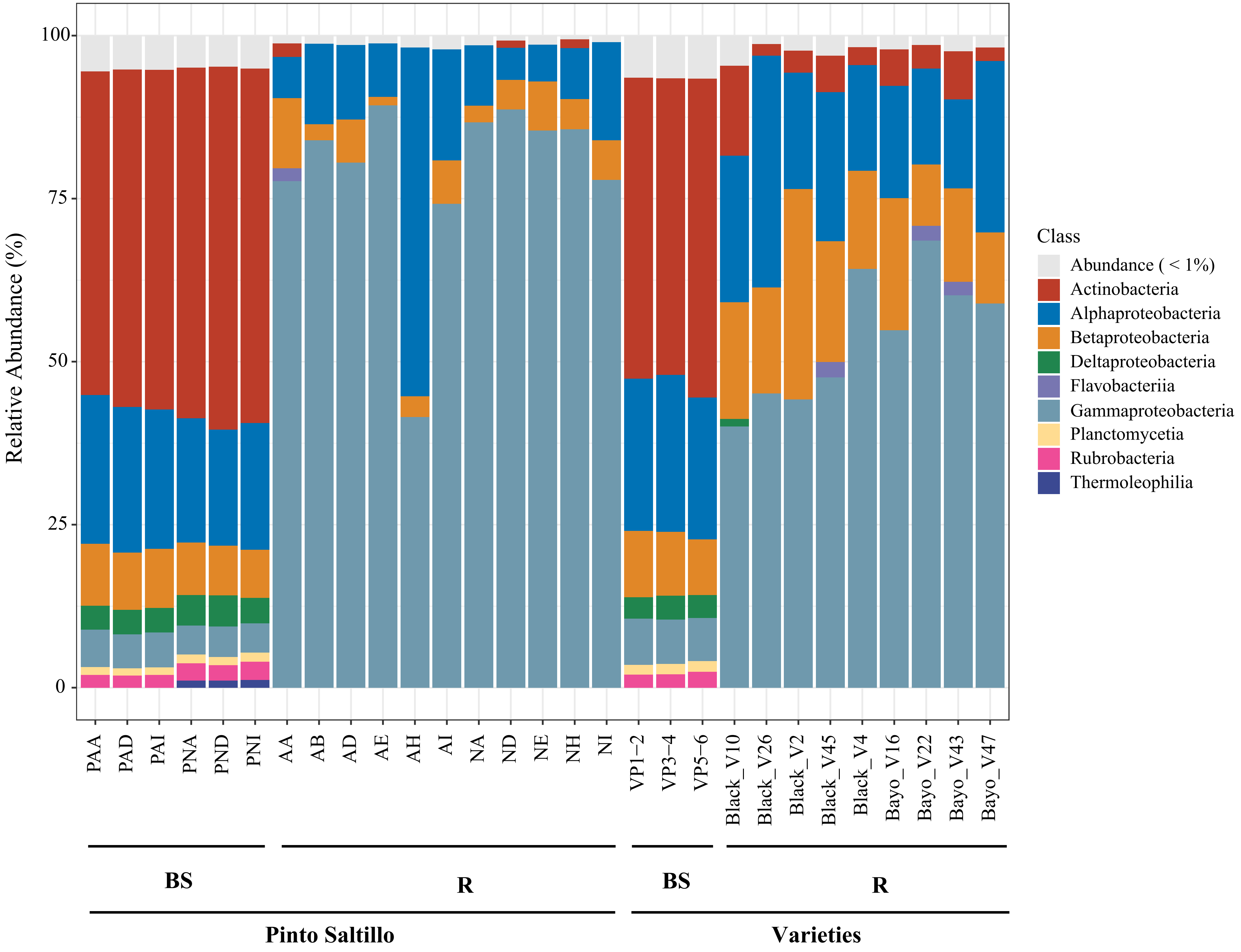

S18 Fig. Relative abundance of bacterial class in bulk soil and rhizosphere. The stacked bars indicate the bacterial classes abundant in bulk soil. Actinobacteria (red), Alpha-Proteobacteria (blue), and Beta-Proteobacteria (orange). Rhizosphere is represented by Gamma-Proteobacteria (light blue) and Alpha-Proteobacteria (blue), and Beta-Proteobacteria (orange). Bacterial classes with less than 1% abundance are shown in light gray.
