# Supplementary material for: The rhizosphere of Phaseolus vulgaris L. cultivars hosts a similar bacterial community in local agricultural soils
Source: PLoS One. 2025 Mar 20;20(3):e0319172. doi: 10.1371/journal.pone.0319172 (PMC11925306; doi:10.1371/journal.pone.0319172)
Supplement: S19 Fig — (A) Rhizobium, (B) Pseudomonas, and (C) Variovorax. (PDF) [file pone.0319172.s020.pdf]

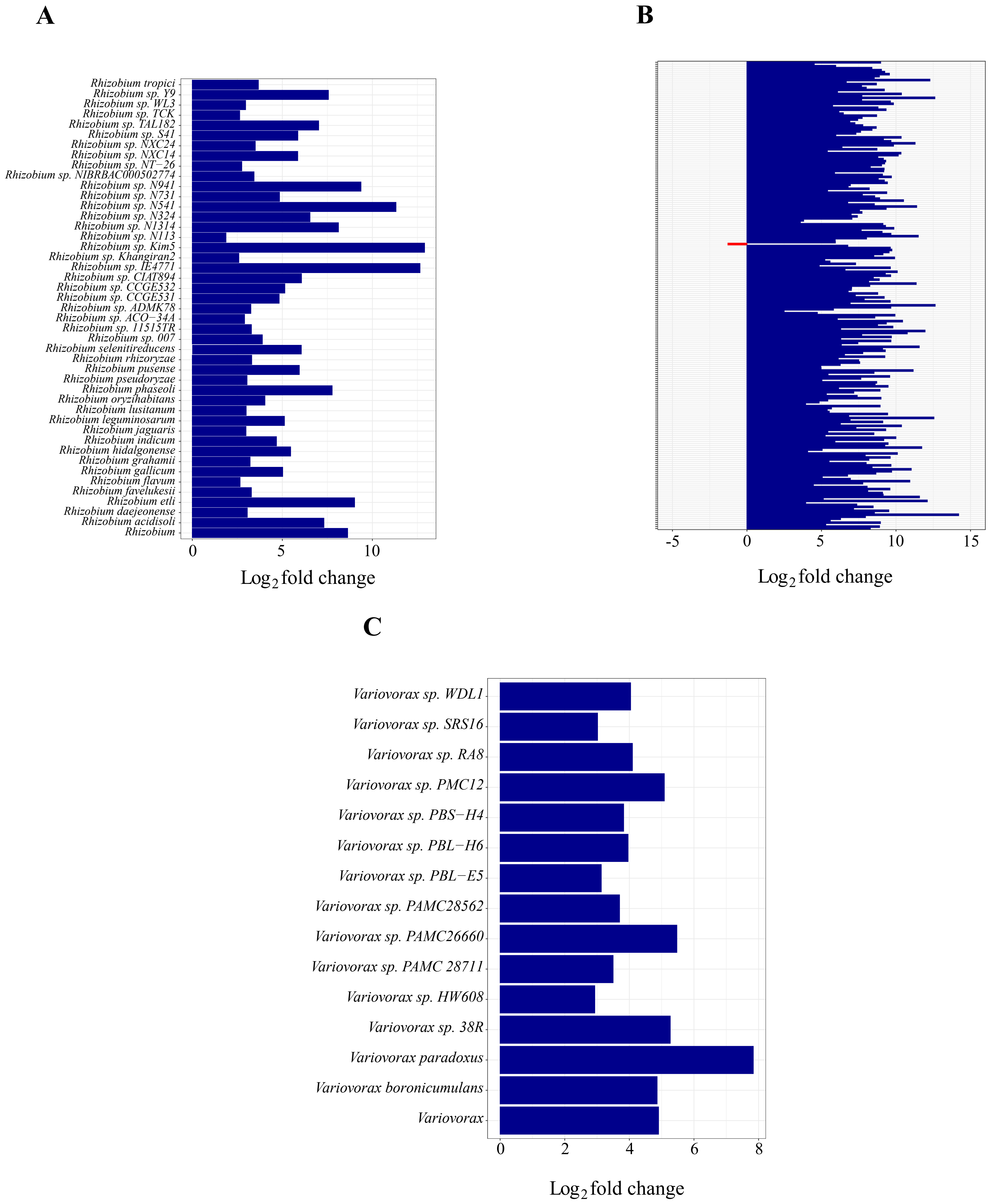

S19 Fig. Number of species in selected enriched genera in the rhizosphere of Pinto Saltillo cultivar. A. Rhizobium, B. Pseudomonas, and C. Variovorax.
