# Supplementary material for: The rhizosphere of Phaseolus vulgaris L. cultivars hosts a similar bacterial community in local agricultural soils
Source: PLoS One. 2025 Mar 20;20(3):e0319172. doi: 10.1371/journal.pone.0319172 (PMC11925306; doi:10.1371/journal.pone.0319172)
Supplement: S20 Fig — Diversity indices calculated from the grouping of bulk soil and rhizosphere metagenomic samples of Black bean (n = 5) and Bayo bean (n = 4). Wilcoxon test is equal to 0.05 for the differences between bulk soil and rhizosphere communities. (PDF) [file pone.0319172.s021.pdf]

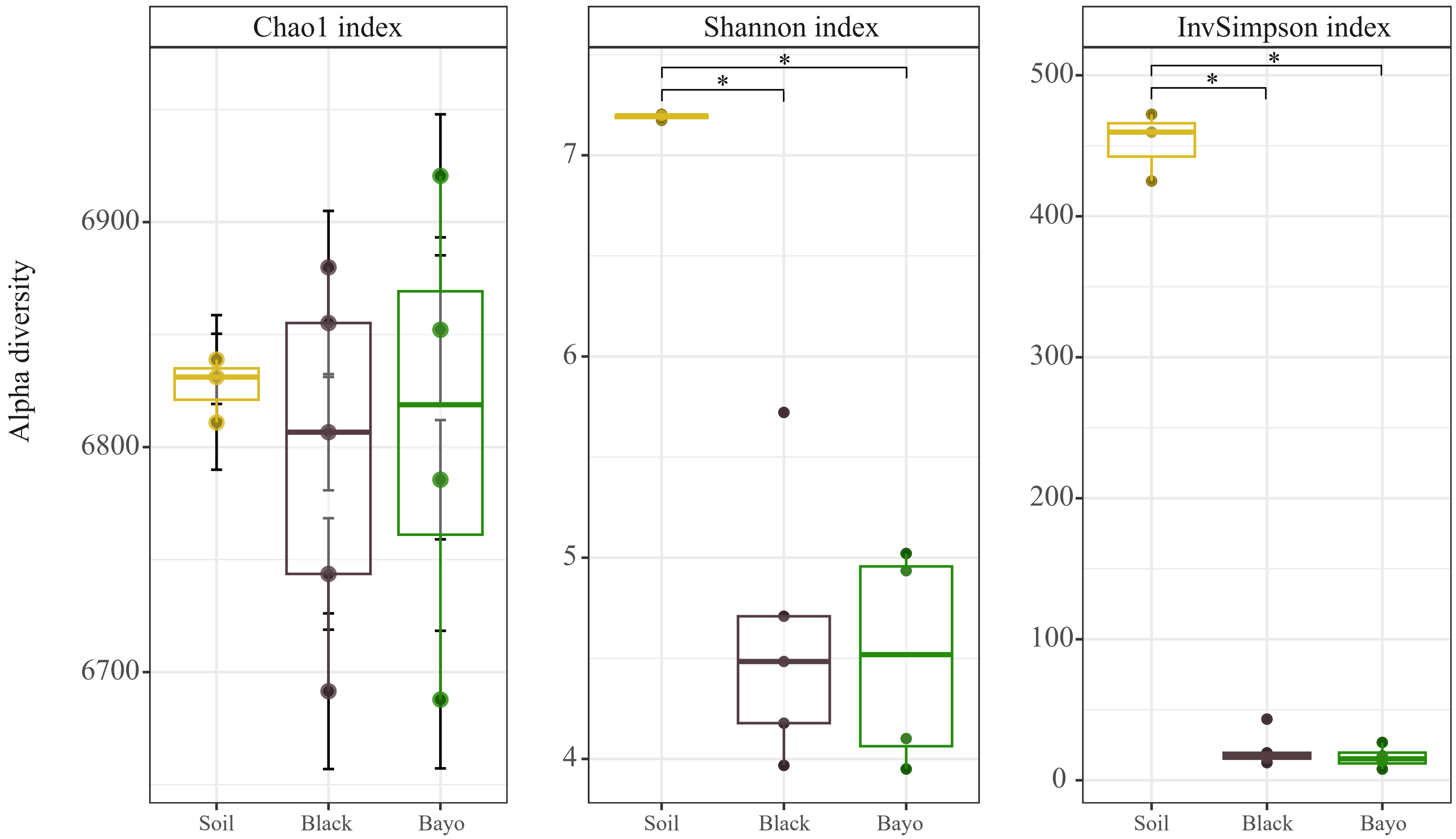

S20 Fig Alpha diversity of Black and Bayo bean cultivars. Diversity indices calculated from the grouping of bulk soil and rhizosphere metagenomic samples of Black bean (n=5) and Bayo bean (n=4). Wilcoxon test is equal to 0.05 for the differences between bulk soil and rhizosphere communities.
