# Supplementary material for: The rhizosphere of Phaseolus vulgaris L. cultivars hosts a similar bacterial community in local agricultural soils
Source: PLoS One. 2025 Mar 20;20(3):e0319172. doi: 10.1371/journal.pone.0319172 (PMC11925306; doi:10.1371/journal.pone.0319172)
Supplement: Attachment — Submitted filename: Rebuttal_letter-3.docx [file pone.0319172.s033.docx]

**Rebuttal letter.**

**Journal Requirements:**

Please review your reference list to ensure that it is complete and correct. If you have cited papers that have been retracted, please include the rationale for doing so in the manuscript text, or remove these references and replace them with relevant current references. Any changes to the reference list should be mentioned in the rebuttal letter that accompanies your revised manuscript. If you need to cite a retracted article, indicate the article’s retracted status in the References list and also include a citation and full reference for the retraction notice.

R. Reference list was revised and updated. Two references were added in response to the reviewer #2 comment on taxonomy (see below)

**6. Review Comments to the Author**

Reviewer #2: Thank you for your efforts in considering all the suggestions. I believe the manuscript has significantly improved as a result.

I have two minor comments for further refinement:

1. When referring to R2 (R-squared), please ensure that the superscript formatting is applied.

R. The notation of R-square was formatted according to the convention, as cited in the manuscript.

2. Kindly review the updated guidelines for referring to bacterial phyla, as outlined in the following resource: NCBI Taxonomy Update on Prokaryotic Phyla.

R. The taxonomy has been actualized according to your comment. The names of the phyla have been modified accordingly throughout the manuscript, figures, and supplementary figures and tables. Table S6 was also modified to include the new taxonomic ranks and its synonyms. Additional lines (l. 199-202) were added to the methodology to account for these changes, in agreement with the NCBI Taxonomy. Two additional references concerning these modifications have also been included.

References included:

39. Pallen MJ. The dynamic history of prokaryotic phyla: discovery, diversity and division. International Journal of Systematic and Evolutionary Microbiology. 2024;74(9):006508. doi:10.1099/ijsem.0.006508.

40. Göker M, Oren A. Valid publication of names of two domains and seven kingdoms of prokaryotes. International Journal of Systematic and Evolutionary Microbiology. 2024; 74(1):006242. doi: 10.1099/ijsem.0.006242
