# Supplementary material for: The rhizosphere of Phaseolus vulgaris L. cultivars hosts a similar bacterial community in local agricultural soils
Source: PLoS One. 2025 Mar 20;20(3):e0319172. doi: 10.1371/journal.pone.0319172 (PMC11925306; doi:10.1371/journal.pone.0319172)
Supplement: Attachment — Submitted filename: Rebuttal_letter.docx [file pone.0319172.s032.docx]

**Review Comments to the Author**

Reviewer #1: I thoroughly enjoyed reading this manuscript. It was well written, justified, and organized and I commend the authors for providing a manuscript in such complete fashion.

However, before I can recommend any detailed changes the authors must provide some crucial information to the validity of the study related to their bioinformatics pipeline and subsequent diversity analyses.

First, for clarification the DNA was extracted from the rhizosphere soil, shotgun sequenced, and then run through a metagenomics pipeline that included using Kraken2 to taxonomically identify the reads. These taxonomic assignments were then used to conduct a diversity analysis of the samples. Is this correct?

R. Thank you for your comments. This is a summary of the experimental design. The taxonomic lower-level taxonomic classification of Kraken2 (species level) was used to model the rarefaction and diversity analysis (l. 185- 215; Supporting Methods)

These next questions will be made under the assumption the above pipeline is correct.

My major questions revolve around the validity of these taxonomic assignments based on WGS/shotgun reads.
1. How accurate/robust is the database from which these genes are taxonomically identified? The reason most people use 16S rRNA as a marker is that it is consistently present across all bacteria and is well annotated for species. Taxonomic ID's made from other functional genes (such as with shotgun metagenomic sequencing), are often much less resolved and thus your results are biased towards organisms with the most representation in the database. Please provide reasoning/justification for this process and evidence of reasonable non-biases for the datbase used.

R. In this study, we aimed to capture a broad range of genomic sequences to determine the diversity of the bacterial taxa. We utilized Kraken2 in our analysis with the PlusPFP database because of its robustness for broad-spectrum taxonomic classification of microbiomes (1-3). PlusPFP encompasses a wide range of microorganisms, expanding upon the Standard Database, which includes archaeal, bacterial, viral, and human sequences from NCBI RefSeq. Furthermore, PlusPGFP incorporated supplementary reference sequences for protozoa, fungi, and plants. We anticipated covering and discarding contaminant sequences from both plants and humans. Additionally, we expected sequence information for eukaryotes (fungi) and viruses; however, they were poorly represented in the metagenomes (see response 2 below) and were not utilized in our analysis. Conversely, the bacterial domain exhibited the highest number of sequences reads and diversity in metagenomes (S2 Table; S3 to S5 Figs.)

Several studies have addressed the performance of Kraken2 using different confidence parameters in simulated and mock databases of various sizes (2, 4). Kraken2 demonstrates efficacy in classifying sequence reads using a large database, similar in size to PlusPFP, at a default confidence score of zero (4). At this confidence score, a satisfactory recall of taxa remained, approximating the optimum of 0.69 (with a confidence score of 0.10). This indicates that these parameters accurately identified true-positive taxa. However, at a default score of zero, the overall performance of F1 is considered suboptimal (4).

Considering these parameters, we evaluated the effect of the default confidence score (zero) and three more stringent confidence score parameters (0.2, 0.4, and 0.6) on a sample of metagenomes derived from the bulk soil and rhizosphere. A consistent decrease in the number of classified sequence reads was observed as the confidence threshold increased from the default value of 0 to 0.6, accompanied by a corresponding increase in unclassified reads (S4 Fig.). In both the bulk soil and rhizosphere, the classified reads assigned to species diminished substantially, along with ~~the~~ diversity parameters (S5 and S6 Figs.). The Shannon index also exhibited low values at a strict confidence level threshold (S6 Fig). . Furthermore, the effect on classified reads was more pronounced in bulk soil than in the rhizosphere. This result led us to conclude that stringent confidence levels did not capture the full diversity of the sample, and significant taxa could be excluded because of their low representation, such as in bulk soil. Consequently, we opted to utilize the default Kraken2 parameters and subsequently examined the changes in abundance between the soil and rhizosphere to assess whether poorly represented taxa in the soil transitioned to abundance in the rhizosphere.

In the second instance, we evaluated the performance of Kraken2 using ribosomal RNA gene databases, specifically GreenGenes and RDP (5). In S7 Fig. shows comparisons of taxonomic assignments made using Plus-PFP, GreenGenes, and RPD databases. A total of 55% (808/1464) of the genera classified using Kraken2-PlusPFP were shared with the Kraken2-RDP database (S7 Fig.). Conversely, the number of classified genera in the GreenGenes database was less than that with RDP and shared approximately 38% (564/1464) with the classification with Kraken2-PlusPFP. Furthermore, the classifications with RDP were sparsely distributed among the metagenomes, in contrast to the homogeneity observed in the distribution of the Kraken 2-PlusPFP assignments across all 29 metagenomes (S7 Fig.).

Therefore, we concluded that the use of Kraken2 with the PlusPFP database provides a robust and diverse taxonomic classification for metagenomic sequences, especially in the bacterial domain. However, the application of stricter confidence parameters significantly reduces the taxonomic diversity detected, which may exclude important taxa with low representation, as observed in soil samples. Therefore, the default parameters of Kraken2 were preferred to avoid the loss of taxonomic diversity. However, the use of ribosomal gene databases (GreenGenes and RDP) with Kraken2 can complement the classification, although they have limitations compared to PlusPFP owing to differences in the coverage and distribution of taxa in the metagenomes. These discrepancies deserve further analysis, and detailed experiments should be performed with combined databases to exhaustively examine bacterial diversity in the soil and rhizosphere (3, 4).

This material and methods are available in the additional file Supporting Methods.

1. Wood, D.E., Lu, J. & Langmead, B. Improved metagenomic analysis with Kraken 2. *Genome Biol* **20**, 257 (2019). <https://doi.org/10.1186/s13059-019-1891-0>
2. Lu J, Rincon N, Wood DE, Breitwieser FP, Pockrandt C, Langmead B, Salzberg SL, Steinegger M. Metagenome analysis using the Kraken software suite. Nat Protoc. 2022 Dec;17(12):2815-2839. doi: 10.1038/s41596-022-00738-y.
3. Edwin NR, Fitzpatrick AH, Brennan F, Abram F, O'Sullivan O. An in-depth evaluation of metagenomic classifiers for soil microbiomes. Environ Microbiome. 2024 Mar 28;19(1):19. doi: 10.1186/s40793-024-00561-w.
4. Wright RJ, Comeau AM, Langille MGI. From defaults to databases: parameter and database choice dramatically impact the performance of metagenomic taxonomic classification tools. Microb Genom. 2023 Mar;9(3):000949. doi: 10.1099/mgen.0.000949. PMID: 36867161; PMCID: PMC10132073
5. Lu J, Salzberg SL. Ultrafast and accurate 16S rRNA microbial community analysis using Kraken 2. Microbiome. 2020 Aug 28;8(1):124. doi: 10.1186/s40168-020-00900-2. PMID: 32859275; PMCID: PMC7455996.

2. Even if this method is valid and supported for taxonomic analysis, you provide no information about organisms other than bacteria, yet your taxonomic assignments were made across bacteria, fungi, protozoa, and plants. Did you remove those non-bacterial sequences prior to analysis, or were they left in? If left in, why do you only talk about the samples as if they contain bacteria? Please confirm or expand upon the methods used to generate "bacteria only" datasets from WGS.

R. We apologize for the inadequate description of this aspect of our research. It is pertinent to clarify (which has now been emphasized in the manuscript, l. 237-252; Supporting Methods) that our focus was on studying the diversity and composition of the bacterial community in bulk soils and in the rhizospheres of common bean cultivars. Consequently, we selected a DNA extraction kit capable of managing a wide range of bacteria but not specifically designed for eukaryotes, such as fungi. DNA samples were obtained using the Qiagen DNeasy PowerSoil Kit (Qiagen). Although the manufacturer stated that the kit is also suitable for fungi, in our experience, it is poorly represented (S4 Table).

In our experiments, a total of 12-14 Gb was sequenced for each metagenomic sample from the bulk soil and the rhizosphere. Most of the classified readings belonged to bacteria (97% and 99% for the bulk soil and rhizosphere, respectively), archaea (0.42, 0.01%), Eukarya (1.2, 0.2%), and viruses (0.02, 0.01%). Bacteria were the most abundant domains and the focus of this study (S4 Table; S3 Fig). Sequence reads other than bacteria, such as eukaryotes, archaea, and viruses, were excluded from further analysis using PhyloSeq.

3. A final question needed answering is the validity of diversity analysis made upon a gene profile that likely contains more than one representative of each individual organism. If organism A is highly abundant (and/or has a large genome), and you count all instances of a reads mapping to organism A, you're going to have a much larger sample of organisms A genes compared to a less abundant, or smaller genome-size organism, thus inflating the prevalence of organisms-A. Please provide justification or more detailed methods regarding how the data was handled in post-processing and bioinformatics.

R. It is acknowledged that several factors may potentially inflate the abundance count in Kraken2. The inflation of kmers in Kraken2 due to the genome length of the organisms likely introduces a bias in the abundance counts. This may also be influenced by the representation of an organism in the database. This issue was addressed by Lu et al. (2009) (6), through Bayesian Re-estimation of Abundance after Classification with Kraken2, or BRACKEN. Furthermore, BRACKEN estimated species despite their close relatedness. We concur with the reviewer that read counts can be assigned as the most represented. To address this, as suggested by Reviewer # 2, we re-analyzed Kraken2 data using BRACKEN. No differences were observed between the composition and abundance of taxa at the genus level between Kraken2 and BRACKEN (S11 Fig.). Additionally, we performed the beta diversity analysis using the abundance matrix of the bulk soil and rhizosphere of Pinto Saltillo (S11B Fig.) calculated with Bracken. This indicates that there were no differences with respect to the same analysis performed with the Kraken2 matrix (Fig. 1B).

6. Lu, J., Breitwieser, F. P., Thielen, P. & Salzberg, S. L. Bracken: estimating species abundance in metagenomics data. PeerJ Comput. Sci. 3, e104 (2017).

Reviewer #2: "The rhizosphere of domestic Phaseolus vulgaris L. cultivars hosts a similar bacterial community in local agricultural lands"

4. The authors have made a valuable contribution to a topic of great significance of local and global relevance, presenting intriguing results in a well-organized manner. However, considering that it is a metagenomic study based only on read-based taxonomic assignment using Kraken2, it appears somewhat simplistic and represents a missed opportunity to leverage the extensive information generated for further analysis. For instance, examining other groups of organisms, such as eukaryotes and viruses, would be valuable. Additionally, enhancing taxonomic assignment by utilizing contigs instead of a read-based approach or conducting functional potential predictions after assembly could provide deeper insights, potentially leading to the reconstruction of genomes from metagenomes. It is important to note that read-based assignments can sometimes be overestimated. Considering the work as it stands and in order to improve quality and validate the results obtained, it is advisable to revisit the methodology to incorporate additional tools and methods that consider the nature of HTS data. Additionally, including the software and packages versions used would enhance transparency. Several suggestions have been proposed to further enhance the overall quality of the manuscript.

R. Thank you for your insightful comment. We revised the manuscript in accordance with your concerns and suggestions. Indeed, we covered the extensive metagenomic information requested and performed compositional analysis of the HTS data according to the suggested methods. We have addressed all these points in the revised version of the manuscript and in the responses to reviewer #1. Versions of bioinformatic tools have been cited.

We conducted analyses of viruses and reconstructed MAGs from the contigs. These components were not included in the manuscript, as we focused exclusively on bacteria.

Another reason for excluding viruses and eukaryotes is the low abundance of these domains in the bulk soil and rhizosphere, as described in response to query 1 (reviewer 1; S3 Fig.; S4 Table). Regarding viruses, metagenomic data did not reveal the full extent of diversity. Rarefaction curves demonstrated that the viral readings were limited (Fig. 1 at the end of this document). A thorough examination of viral diversity warrants an experimental approach for the enrichment of viral readings. Santos-Medellín et al. published a comparison of viral metagenomic reads versus enrichment of the viral component in the rhizosphere of tomato (7). This does not imply that we disregarded the viral components. Our limited data indicate that viral diversity is correlated with the most abundant bacterial genera in samples. For instance, viruses associated with *Pseudomonas, Rhizobium, and Flavobacterium*, the most abundant genera classified by Kraken2, were the most abundant phages detected in the viral metagenomic viral fraction. However, these findings are insufficient to draw conclusions regarding the interplay between viruses and bacteria. Ongoing experiments focus on obtaining a more suitable representation of viruses using both viral cultivation and viral metagenomic enrichment approaches.

MAGs were reconstructed from contigs assembled from metagenomic readings; however, much of this work is still in progress. MAGs were consistent with the most abundant taxa determined by Kraken2 (Figure 2 at the end of this document). Here, we present some examples of MAGs coverage. We intended to describe the functional structure of the bulk soil and rhizosphere in another study, including details of the reconstruction of MAGs.

7. Santos-Medellin C, Zinke LA, Ter Horst AM, Gelardi DL, Parikh SJ, Emerson JB. Viromes outperform total metagenomes in revealing the spatiotemporal patterns of agricultural soil viral communities. ISME J. 2021 Jul;15(7):1956-1970. doi: 10.1038/s41396-021-00897-y.

Abstract:
• The abstract is well-presented and organized.

R. Thanks.

Introduction:
• L59 and L62: Fabaceae are a family. Only genera and species must be in italic. Also, this line should be rewritten considering this, because Fabaceae are not species.

R. You are correct, and this phrase is incorrect. It was modified as follows: “*Nitrogen fixation symbiosis between Rhizobium and diverse leguminous species of the Fabaceae family is an outstanding example of the close relationship between plant roots and bacteria’* (l. 65- 67).

• The introduction should include information on Cultivation History and its impact on bacterial communities.

R. A new paragraph introducing the effects of cultivation history and other agricultural factors is now in the manuscript version (l. 79- 93).

Material and methods
• L136: More details about physical and chemical characterization are needed in the methods section.

R. We have now included a reference to the methods according to the Mexican Official Standard (NOM-021-RECNAT-2000). This is in l. l60- 162: “The physicochemical properties of the soil were analyzed at the INIFAP Zacatecas Soil Laboratory following the protocols outlined in the Mexican Official Standard (NOM-021-RECNAT-2000), which provides specifications for soil fertility, health, and classification”.

• L145: Is this analysis a PCA or a PCoA? Additionally, to assess differences between sites, a statistical test, such as a t-test or Wilcoxon test (depending on the normality of the data), could be employed.

R. It is indeed a PCA. We performed PERMANOVA using Euclidian distances. It indicates that there no statistically significant differences between A (Agricole) and N (Non-agricola) sample, regarding the physical chemical properties.

*SumOfSqs=958.76, R2=0.64161, F=16.112, pval=0.002*

*Residual SumOfSqs=535.55, Residual R2=0.35839.*

This information has now been added in the S2 Fig.

• L159: Sequences “were” instead of “was”.

R. Corrected.

• L162: If the PlusPFP database was utilized, it would be important to clarify why other groups of organisms, such as viruses and eukaryotes, were not included in the analysis, particularly given that a metagenomic read-based taxonomic assignment was performed.

R. The reasons for not including viruses and fungi were given in lines above (first response) and in the responses to reviewer # 1. Virus and eukaryote sequence readings were underrepresented in shotgun metagenomic data (S3 Fig.; S4 Table; Fig. 1 this document). Therefore, they were excluded from the study using the Phyloseq command lines.

• L164: Considering that depth of sequencing could potentially be an issue; it is unclear whether a rarefaction or any type of normalization was conducted or if only rarefaction curves were generated. Providing a repository with scripts and analyses would greatly assist readers in understanding the procedures, methods and statistical analyses employed.

R. The sequencing depth was substantial for a complex community, such as the soil and rhizosphere (approximately 12-14 Gb; S4 Table; S10 Figure). We conducted a comparison of the sequencing depth for rhizosphere and bulk soil in other cultivated plants, including tomato, cucumber, maize, wheat, beans (other study), and the model plant *Arabidopsis*, with our metagenomic samples (S9A Fig). The number of sequenced metagenomic reads in these plants was significantly lower than that in our study (six–eight times less); however, the calculated species richness was nearly equivalent. Upon normalizing all the sequence reads of the metagenomes studied here to the same number of sequences reads of the other plants, we observed an asymptote in all metagenomes, indicating adequate coverage of diversity (S9B Fig). Given that we collected an average of 80 million reads in average, we determined that normalization of the data was unnecessary.

A repository of the bioinformatics and statistics methodology associated with this study is currently available at GitHub (<https://github.com/vgonzal54/common-bean-rhizosphere>). Moreover, we have included detailed descriptions in the Materials and Methods section, Supporting Methods, and figures.

• Also, when Kraken2 is used, it is recommended to re-estimate the abundance with Bracken program (Lu et al., 2017).
- Lu, J., Breitwieser, F. P., Thielen, P., & Salzberg, S. L. (2017). Bracken: estimating species abundance in metagenomics data. PeerJ Computer Science, 3, e104.

R. Thank you for this suggestion. We re-analyzed the Kraken2 data using BRACKEN. We did not observe differences between the composition and abundance of taxa at the genus level between Kraken2 and Bracken (S11 Fig.). Furthermore, we performed beta diversity analysis using the abundance matrix of the bulk soil and rhizosphere of Pinto Saltillo (S11B Fig) calculated using Bracken. This indicates that there were no differences with respect to the same analysis performed with the Kraken2 matrix (Fig. 1B).

• L167-L170: I strongly recommend taking into consideration the inherent characteristics of High-Throughput Sequencing (HTS) data, which can be both spurious and compositional due to the arbitrary total imposed by the instrument (Gloor et al., 2017). To address this issue, it is advisable to employ methods such as differential abundance tests, which can vary depending on the specifics of your research and the nature of your data (Nearing et al., 2022). Properly managing the data before hypothesis testing is essential to ensure the robustness of your results.
- Gloor, G. B., Macklaim, J. M., Pawlowsky-Glahn, V., & Egozcue, J. J. (2017). Microbiome Datasets Are Compositional: And This Is Not Optional. Frontiers in Microbiology, 8, 2224. <https://www.frontiersin.org/article/10.3389/fmicb.2017.02224>
- Nearing, J. T., Douglas, G. M., Hayes, M. G., MacDonald, J., Desai, D. K., Allward, N., Jones, C. M. A., Wright, R. J., Dhanani, A. S., Comeau, A. M., & Langille, M. G. I. (2022). Microbiome differential abundance methods produce different results across 38 datasets. Nature Communications, 13(1). <https://doi.org/10.1038/s41467-022-28034-z>

R. We welcome the reviewer’s suggestion and agree that the relative abundance matrix used in DESeq2 could not be reliable for comparisons between samples and did not account for the restriction of the high-throughput sequencing outcomes. Therefore, following the recommendation, we first transformed the read count in the metagenomes using centered log-ratio transformation (CLR) to compute abundance changes without bias in the relative abundance measure. Using 1472 taxa at the genus level (classified by Kraken2), we determined that 80 out of 1472 samples had zero-percentage less than 0.1%. Then, using the CLR matrix, we performed PCoA analysis with the Aitchinson distance matrix to examine the differences between bulk soil and rhizosphere bacterial community composition and between pairs of rhizosphere communities of the three cultivars (Pinto Saltillo, Bayo, and Black cultivar; S10 Fig.). Rhizosphere communities significantly differed from soil communities, as indicated by PERMANOVA (S8 Table); p=0.001; F=8.1; R^2^=0.21), whereas the Pinto Saltillo rhizosphere communities differed from the bacterial rhizosphere of Bayo (p=0.25) and black bean cultivars (P=0.21; F=2.4; R^2^=0.32). Furthermore, the rhizosphere communities of Bayo and Black beans did not differ significantly from each other (S8 Table). Similar conclusions were drawn from the data analyzed using the relative abundance data described in the manuscript. Hence, the compositional approach strengthened the previous DESeq2 analysis results.

Another concern of our study was the differential abundance estimated using DESeq2. As discussed above, the metagenomic dataset included all Kraken2 classified taxa. We then obtained a broad spectrum of diversity, as was our objective, but at the cost of including low-abundance, rare, and false-positive taxa. Consequently, this may result in data overdispersion. However, in the metagenome studied, beta-dispersion was reasonably low and did not affect PERMANOVAS when comparing Pinto Saltillo rhizosphere and bulk soil communities (S12 and S13 Figs.). Therefore, we considered that DEseq could manage the comparisons of differential abundances shown in Figures 1B and 4B without the need for data normalization. Indeed, the relatively low zero counts in the metagenomes allowed us to use the DESeq2.

However, as suggested, we performed ANCOMBC with the CLR abundance matrix to account for the compositional structure in the differential abundance estimates. The assessment of the change in taxa (at genus level) from soil to the rhizosphere in the three common bean cultivars with ANCOMBC indicate that abundant taxa, upper than two log-fold change (2-LFC) were included in the DESeq2 group of similar LFC (S14 and S15 Figs.). These results indicate that, although ANCONBC is more conservative and identifies less abundant taxa than DESeq2, the conclusions in the manuscript remain valid.

• Also, cite and detail packages of R used (packages instead of programs) with versions and properly citations.

R. Thank you for pointing this out. We have added references and proper descriptions of the bioinformatics and statistical packages.

• L171: It is advisable to use the term “statistical tests” rather than “statistics tests” to ensure clarity and precision

R. Corrected.

Results
• L185: Species and genera names always in italics.

R. Revised and corrected throughout the manuscript.

• L202: Are these OTUs? According to the literature, OTUs are defined as clusters of sequencing reads that differ by less than a fixed dissimilarity threshold (Callahan et al., 2017). Furthermore, Kraken2 is not a clustering algorithm program but rather a sequence classifier. It utilizes k-mers and “de Bruijn” graphs to efficiently classify sequences by comparing them to a reference database of known sequences.
⎫ Callahan, B. J., McMurdie, P. J., & Holmes, S. P. (2017). Exact sequence variants should replace operational taxonomic units in marker-gene data analysis. The ISME journal, 11(12), 2639-2643.

R. It is an unfortunate misconception from us to equate OTUs with the taxonomic classification by Kraken2. The manuscript has been meticulously revised to indicate that we have assigned taxa at the species and genus levels, not OTUs. Kraken2 classifies sequences using the Lowest Common Ancestor concept to capture uncertain assignations to the closest taxonomic ranks. Moreover, Kraken2 can be classified at the species level. In our study, we observed that Kraken2 distinguished multiple species within abundant genera, such as *Pseudomonas, Rhizobium,* and *Pantoea* (S19 Fig.). Therefore, the most abundant genera comprise an assemblage of different species, as classified by Kraken2.

• What taxonomic unit or level does Kraken2 provide? It is important to standardize this terminology throughout the text for consistency.

Kraken2 classifies sequence reads at all taxonomic hierarchies (Domain, Phylum, Class, Order, Family, Genus, and Species) derived from the NCBI taxonomy. The genome sequences in the Kraken2 database were constructed and indexed as k-mer profiles. Kraken2 subsequently analyzed the k-mers of the sequence readings and attempted to match them with the database. Kraken assigned a taxonomic rank to the sequence reads with the highest number of matched k-mers to taxa in the database. The absence of matches was interpreted as being unclassified. Kraken2 assigns sequences to the lowest common ancestor (LCA) of matching reference sequences in its database. The resolution of the classification is dependent on the completeness and specificity of the database as well as the quality of the input sequences. Kraken enables classification to be performed at any taxonomic rank. Several factors can influence classification, such as ambiguous and low-abundance k-mers. Kraken2 classified these sequences as taxon rankings as determined by LCA.

We make clear at which taxonomic hierarchy of Kraken2 we are referring along the manuscript

• In Figure 1, the point that is distance to the others could be potentially an outlier? Consider the possibility and how to manage this type of data. Also, given the findings presented, it is recommended to conduct a betadisper analysis to validate the results of the perMANOVA. This additional analysis could provide insights into the dispersion of diversity within the groups.

This sample was AH, as indicated in line 207. As recommended, we conducted a betadisperser test to assess the homogeneity of variances in the data presented in Figure 1 (S12 Fig.). Rhizosphere samples and bulk soil of Pinto Saltillo exhibited a moderate distance from the median (or centroid). The p-value of Levene's test was 0.1675, indicating no significant differences in the variances within the group (S12E Fig.). The removal of the AH sample did not significantly affect the dispersion values (p = 0.2185). Consequently, the differences are less likely to be confounded by heterogeneity in variances, and the observed difference in the rhizosphere communities relative to the bulk soil can be attributed to the large variances in composition that PCoA analysis show as statistically evaluated by PERMANOVA.

• L209: The results of the PERMANOVA should be presented in detail, including not only the p-value but also the statistic, degrees of freedom, etc. This information could also be included in detail in a supplementary table.

Details of the statistical analysis are presented in the following tables. Other statistics such as betadispersion and Levene´s tests are in S12 and S33 Figs.

S4 Table**.** Statistics of metagenomic sequences analysis, and distribution of reads in domains determined by Kraken2.

S5 Table. PERMANOVA statistics for beta diversity of bulk soil and rhizosphere bacterial communities, in agriculture (A) and non-agriculture (N) plots, sown with Pinto Saltillo Cultivar. PCoA performed with Bray Curtis distances.

S6 Table. Differential abundance (DESeq2) of taxa at genus level in bulk soil and rhizosphere.

S7 Table. PERMANOVA statistics for beta diversity of bulk soil and rhizosphere bacterial communities, in agriculture (A) and non-agriculture (N) plots, sown with Pinto Saltillo. PCoA performed with Bray Curtis distances.

S8 Table. PERMANOVA statistics for beta diversity of bulk soil and rhizosphere bacterial communities, in agriculture (A) and non-agriculture (N) plots, sown with Pinto Saltillo Black, and Bayo bean cultivars cultivars. Abundance tables were calculated with CLR and beta diversity with the Aitchinson distance.

S9 Table. Shared taxa at genus level in the Venn diagram of Fig. 5. Only genera enriched at LogFoldChange (LFC) >2 in Pinto Saltillo, Black, and Bayo cultivars are shown.

• In figure 2 and 4, The colors are similar in some genera, making it difficult to distinguish between them.

R. The colors have been modified to highlight these differences.

• L226: All genus and species names must be italicized throughout the text. This error is consistent, as some are italicized while others are not.

R. All these names have been corrected throughout the manuscript.

• L240: DESeq2 results are in Figure 2.

R. Corrected

• Results of the effect cultivation history in perMANOVA, alpha diversity and DESeq2 results are not presented. I suggest that these not significant values and results should be presented in order to express and discuss whether cultivation history has relevance or not in this study.

R. As suggested, the PERMANOVA and statistical tests of the effect of cultivation history on bacterial communities are now presented.

a) The Wilcoxon test for inter-site pairs of samples for N (without cultivation history) and A (cultivation history) for bulk soil and rhizosphere are presented in modified Figure 1.

b) PERMANOVAS of the Bray Curtis are indicated in the legend of Figure 1B and in the new supplementary table (S5 Table).

c) DESeq2 differential abundance analysis was conducted separately for A (agricultural site) and N (non-agriculture) in relation to the bulk soil (S16 Fig.). The results indicated that approximately 73% of the taxa exhibited altered abundance in the transition from the bulk soil to the rhizosphere in Pinto Saltillo at sites A and N. Furthermore, a distinct group of species at each site demonstrated changes in the presence of the plant, contingent on the site. These findings have been incorporated into the text (section “Shifts in taxa composition from the bulk soil to the rhizosphere”) in lines 287-297 of the manuscript.

• L286: Figure S3 does not show data about Black and Bayo cultivars.

R. Figure S3 was complemented with Black and Bayo information. It is now S16 Fig.

• In Figure 4B, it is evident that bulk soils are similar regardless of the cultivar type. However, this is not the case in the rhizosphere, where Pinto Saltillo samples show notable differences compared to the Black and Bayo samples. This raises concerns about potential confounding effects. For instance, was the impact of sampling time or seasonality accounted for, given that samples were taken in two different years and months? Additionally, were the sequences processed in two separate batches? (Sequencing and bioinformatic analyses) These factors could introduce confounding variables that might explain the apparent differences, especially since it was noted earlier that the abundance of genera was relatively similar across cultivars. This also applies to perMANOVA and DESeq2 analyses.

R. We acknowledge that these differences may have been caused by confounding factors. Fig 4B shows differences in the rhizosphere between Pinto Saltillo and the other cultivars but similarities between Black and Bayo rhizosphere bacterial communities. This experiment refers to the taxa (genera) that increase in abundance from the soil to the rhizosphere by at least two-fold. A careful review of the statements made about “similar bacterial communities” was carried out, and the text was modified considering the factors that may confound these conclusions, avoiding overstating.

The experiments described here were performed *in situ* in an agricultural field, which differs from other reports performed in greenhouses (references). Plants of the common bean cultivars Black and Bayo were not available when Pinto Saltillo was ready for collection. In this sense, samples of Pinto Saltillo could not be compared directly with Black and Bayo beans because of the differences in collecting time and processing, as you rightly comment. This is now recognized in the corresponding section of the Results and Discussion section.

Discussion
• The overall structure of the discussion is well-organized.

R. Thank you.

• L320: “Our findings indicate that the diversity and structure of the rhizosphere bacterial community are predominantly influenced by the bulk soil bacterial community, and to a lesser extent, by the specific bean variety.” This statement is unclear in terms of how the results support it. Additionally, it seems inconsistent with the statement in L341: “This study observed no significant differences in bacterial diversity across bulk soils of varying physicochemical compositions and cultivation histories.” Please clarify how the data support the conclusion that the rhizosphere is predominantly influenced by the bulk soil bacterial community, especially in light of the observation of no significant differences in bulk soil diversity.

R. Thank you for the observation. This point is insufficiently described in the discussion. Thus, we added new paragraphs to support the conclusion (l. 411-415; l. 435- 465), and three original paragraphs were moved to follow the narrative in a logical fashion. We hope that these modifications clarify the mentioned statements.

• L396: The term “improved microbiome” is vague. Does it refer to greater richness or diversity, or is it about promoting the presence of specific types of bacteria? It would be helpful to define what is meant by "improved microbiome" in this context.

R. We agree, the term “improved microbiome” is incorrect for the intended meaning. The phrase was modified according to the comment as follows: “*It is less apparent what are the effects of plant hybridization, genetic manipulation techniques, and historical domestication practices on the rhizosphere microbiome diversity and function of common bean cultivars” (l. 705-708).*

**Figures.**

 Viral metagenomic contigs.

**Figure 1.** A, Rarefaction curves of the viral OTUs**.** Viral metagenomic reads were assembled into contigs using MegaHIT (8). Viral contigs were searched using Vibrant and classified as Kaiju (9). B Viral associations with genera from the rhizosphere. Counts represent the number of viral OTUS per bacterial genus.

8. Li, D., Liu, C-M., Luo, R., Sadakane, K., and Lam, T-W., (2015) MEGAHIT: An ultra-fast single-node solution for large and complex metagenomics assembly via succinct de Bruijn graph. *Bioinformatics*, doi: 10.1093/bioinformatics/btv033.
9. [Menzel, P. et al. (2016) Fast and sensitive taxonomic classification for metagenomics with Kaiju. *Nat. Commun.* 7:11257](http://www.nature.com/ncomms/2016/160413/ncomms11257/full/ncomms11257.html)

A

B

Figure 2. Ten examples of Metagenome Assembled Genomes (MAGs) recovered from the rhizosphere metagenomes of common bean cultivars. Coverage (A) and main features (B).
